# Psychometric properties of a short knowledge test to evaluate awareness of external ventricular drain care practices among Chinese nurses: a Rasch model analysis of an efficient 7-item self-reported assessment tool

**DOI:** 10.3389/fpubh.2026.1963782

**Published:** 2026-10-16

**Authors:** Linlin Wen, Yuanyuan Sun, Xue Du

**Affiliations:** Department of Hematology, The First Affiliated Hospital of Jinzhou Medical University, Jinzhou, China

**Keywords:** external ventricular drainage, nurses, psychometrics, Rasch model, self-reported knowledge

## Abstract

**Background:**

External ventricular drainage (EVD) is an important intervention in the management of patients with severe neurological conditions, and standardized nursing care is essential for preventing complications and ensuring patient safety. Nurses’ knowledge of recommended EVD care practices is an important foundation for safe and evidence-based care. However, existing instruments for assessing EVD-related nursing knowledge are often lengthy or have limited psychometric validation, which may restrict their use in routine clinical assessment and large-scale training. Therefore, a brief, reliable, and psychometrically robust instrument is needed to efficiently assess nurses’ knowledge of EVD care practices and identify areas requiring targeted education.

**Aim:**

To translate and culturally adapt the Short Knowledge Test to Evaluate Awareness of External Ventricular Drain Care Practices and evaluate its psychometric properties among Chinese nurses using the Rasch model, thereby providing a reliable tool for the rapid assessment of nurses’ self-reported knowledge of recommended EVD care practices and the identification of targeted training needs.

**Method:**

The original scale was translated and culturally adapted into Chinese using the Brislin forward–backward translation method, resulting in the Chinese version of the Short Knowledge Test to Evaluate Awareness of External Ventricular Drain Care Practices (C-SKT-EVD). A cross-sectional survey was conducted among clinical nurses recruited from First Affiliated Hospital of Jinzhou Medical University, Liaoning Province, mainland China. 242 valid participants included in the final analysis. Descriptive statistics were performed using SPSS version 26.0, and the psychometric properties of the scale were evaluated using the Rasch Rating Scale Model in Winsteps version 3.72.3, focusing on unidimensionality, item fit, category functioning, item–person distribution, test information function, separation indices and reliability, and differential item functioning (DIF).

**Results:**

The empirical variance explained by the measures was 55.1%, and the eigenvalue of the first contrast was 1.4, supporting the unidimensionality of the C-SKT-EVD. The Infit mean-square (MNSQ) values for the seven items ranged from 0.94 to 1.16, while the Outfit MNSQ values ranged from 0.94 to 1.07, all indicating acceptable fit to the Rasch model. The point-measure correlation coefficients (PT-Measure Corr.) ranged from 0.63 to 0.70, suggesting good alignment between the items and the underlying latent construct. The observed average measures across the five rating categories increased monotonically, and the category thresholds were ordered sequentially. The category probability curves also showed clearly defined peaks, indicating that the rating categories functioned well and that no category collapsing was required. The item difficulty distribution was generally well matched to the respondents’ levels of self-reported EVD care knowledge, and the test information function indicated higher measurement precision around the average level of the measured construct, with relatively less information provided for respondents with extremely high or low levels of the latent trait.

**Conclusion:**

The C-SKT-EVD demonstrated generally acceptable Rasch measurement properties in the present sample of Chinese nurses, providing preliminary evidence for its use as a brief screening and educational assessment tool for self-reported knowledge of recommended EVD care practices. However, these findings do not establish comprehensive validity or objective clinical competence. Further multicenter studies in diverse nursing populations are needed to confirm its broader applicability.

## Introduction

External ventricular drainage (EVD) is a core invasive intervention in neurocritical care and is widely used in critically ill patients with conditions such as obstructive hydrocephalus, intraventricular hemorrhage, traumatic intracranial hypertension, and subarachnoid hemorrhage. By enabling continuous cerebrospinal fluid drainage and intracranial pressure monitoring, EVD plays a critical role in the management of acute neurological conditions and the prevention of secondary brain injury (1, 2). The safe management of EVD requires standardized, evidence-based nursing practices throughout the entire care process, including strict aseptic technique, dynamic monitoring of cerebrospinal fluid drainage, accurate leveling of the drainage system, and early recognition of potential complications (3). Inadequate EVD management may result in serious adverse events, including ventriculitis, catheter obstruction, catheter dislodgement, and overdrainage, which may prolong hospitalization, increase the risk of morbidity and mortality, and impose an additional healthcare burden (2). Nurses are the primary healthcare professionals responsible for providing continuous, 24-h care to patients with EVDs. Their knowledge of EVD care practices and ability to implement standardized nursing procedures are therefore essential for preventing complications and ensuring patient safety. Systematically assessing nurses’ knowledge of EVD care practices and accurately identifying knowledge gaps are important prerequisites for developing targeted in-service education, standardizing nursing procedures across clinical settings, and implementing evidence-based care bundles (4). Importantly, assessment of EVD-related nursing knowledge should be distinguished from direct evaluation of clinical competence, as knowledge represents an important but not sufficient component of safe clinical practice.

Despite the importance of standardized EVD care, nurses’ knowledge and clinical experience related to EVD management may vary across different clinical settings (5). Nurses working in specialized neurosurgical intensive care units may have more frequent exposure to patients with EVDs, whereas nurses in emergency departments and general medical or surgical wards may have fewer opportunities to provide EVD-related care (6). In addition, differences in institutional training programs and local clinical protocols may contribute to variations in nurses’ understanding of key aspects of EVD care. A recent multicenter study involving neurosurgical nurses from 10 hospitals in China identified considerable differences in nurses’ knowledge, attitudes, and practices regarding EVD management, highlighting the need for standardized education and systematic assessment of EVD-related knowledge (7).

Several instruments have been developed to assess nurses’ knowledge and practices related to EVD care, but their clinical applicability and psychometric evaluation warrant further consideration. For example, Chen et al. recently developed multiple items questionnaire based on the knowledge–attitude–practice (KAP) framework to assess nurses’ knowledge, attitudes, and practices regarding adult EVD care (8). Although the questionnaire demonstrated satisfactory reliability and validity, its relatively large number of items may limit its feasibility for rapid knowledge screening, point-of-care assessment, and repeated evaluation in routine clinical settings, particularly among nurses with heavy workloads and limited time for completing lengthy assessments. In addition, some EVD-related questionnaires have been developed for specific studies or local clinical settings, which may limit their comparability and generalizability across healthcare institutions. Furthermore, most existing instruments have been evaluated primarily using classical test theory (CTT), including exploratory factor analysis (EFA), confirmatory factor analysis (CFA), and Cronbach’s alpha (9–13). Although these methods provide important evidence regarding overall scale structure and internal consistency, they offer limited information about individual item functioning, the match between item difficulty and respondent ability, and potential measurement bias across different subgroups. Therefore, there is a need for a brief instrument that combines practical feasibility with rigorous psychometric evaluation to efficiently assess nurses’ knowledge of EVD care practices and identify specific areas requiring targeted education. The EVD system is characterized by its complex structure, multiple management procedures, and high risk of complications. Nurses’ professional knowledge and standardized nursing practices are directly associated with the effective functioning of the drainage system and patient safety (14).

Erbas et al. developed a 7-item brief knowledge assessment tool for EVD care and conducted preliminary psychometric evaluation of its construct validity and internal consistency using exploratory factor analysis and confirmatory factor analysis (15). The tool demonstrated a unidimensional structure, a concise item set, and excellent internal consistency (Cronbach’s *α* = 0.915), indicating its potential for the rapid assessment of nurses’ knowledge of EVD care in clinical practice. However, because the instrument uses graded agreement with factually oriented statements rather than objectively scored correct/incorrect responses, its scores are more appropriately understood as reflecting a graded level of self-reported knowledge and awareness of recommended EVD care practices. The tool was developed based on a sample of nurses in Türkiye, whose language, nursing practices, and clinical management protocols may differ from those of the Chinese healthcare context. The tool has not yet undergone systematic cross-cultural adaptation and validation in China, and its applicability and measurement stability among Chinese nurses remain to be established. In addition, previous research has primarily relied on classical test theory (CTT) for psychometric evaluation, with relatively limited assessment of item difficulty, person ability, and measurement fairness across different population groups. Therefore, more refined modern measurement approaches are warranted to further evaluate the measurement properties of this tool among Chinese nurses.

As an important model within item response theory (IRT), the Rasch model allows person ability and item difficulty to be estimated on the same measurement scale. It also provides a comprehensive evaluation of measurement quality from multiple perspectives, including unidimensionality, item fit, the distribution of item difficulty and person ability, and differential item functioning (DIF), thereby providing objective evidence regarding the structural validity, measurement precision, and measurement fairness of an instrument across different groups (16). In the present study, the Rasch person measure is interpreted as the latent level of self-reported knowledge reflected by respondents’ graded endorsement of the factually oriented EVD care statements, rather than as an objectively verified level of factual knowledge or clinical competence. Accordingly, applying the Rasch model to the localization and validation of an EVD nursing knowledge assessment tool may provide further insight into its item-level measurement characteristics and complement traditional psychometric approaches by offering more detailed evidence at the item level.

Therefore, this study aimed to translate and culturally adapt the 7-item brief EVD nursing knowledge assessment tool developed by Erbas et al. and to systematically evaluate its psychometric properties among Chinese nurses using the Rasch model, with a particular focus on unidimensionality, item fit, the distribution of item difficulty and person ability, measurement precision, and DIF. This study sought to provide a concise, efficient, and psychometrically sound tool for the rapid assessment of nurses’ self-reported knowledge of recommended EVD care practices in China, thereby facilitating the identification of perceived knowledge gaps among clinical nurses and providing a basis for targeted specialty nursing education, EVD nursing quality improvement, and the standardization of neurocritical care nursing practice.

## Materials and methods

### Translation and cross-cultural adaptation

This study strictly followed the classical Brislin forward–backward translation model and incorporated internationally accepted guidelines for the cross-cultural adaptation of nursing measurement instruments.

The original Short Knowledge Test to Evaluate Awareness of External Ventricular Drain Care Practices was developed by Kadriye Erbas, MS, and colleagues in Türkiye. The original item pool was generated based on a review of the relevant literature and consultation with experts in EVD care. The preliminary items were subsequently evaluated and refined through psychometric testing, resulting in a final seven-item instrument. The items addressed key aspects of EVD care, including cerebrospinal fluid specimen handling and collection, drainage bag replacement, EVD system leveling and positioning, and identification of EVD lines. The original development study reported the psychometric properties of the resulting instrument and established the basis for its use in assessing nurses’ knowledge and awareness of EVD care practices. The present study did not develop a new item pool or independently select items from clinical guidelines. Instead, the seven-item original instrument served as the conceptual and content basis for the Chinese version. The present study focused on forward–backward translation, cross-cultural adaptation, and psychometric evaluation among Chinese nurses. Therefore, the original item content and underlying construct were retained, while minor linguistic and contextual modifications were made to ensure semantic clarity and appropriateness in the Chinese clinical context. The translation and adaptation process comprised three major stages (17): (1) translation and synthesis, (2) expert review and cultural adaptation, and (3) pilot testing and final revision. The process included forward translation, back-translation, expert review, item modification, and pilot testing to complete the Chinese translation and cultural adaptation of the instrument. The specific procedures are described below (Figure 1).

**Figure 1:**
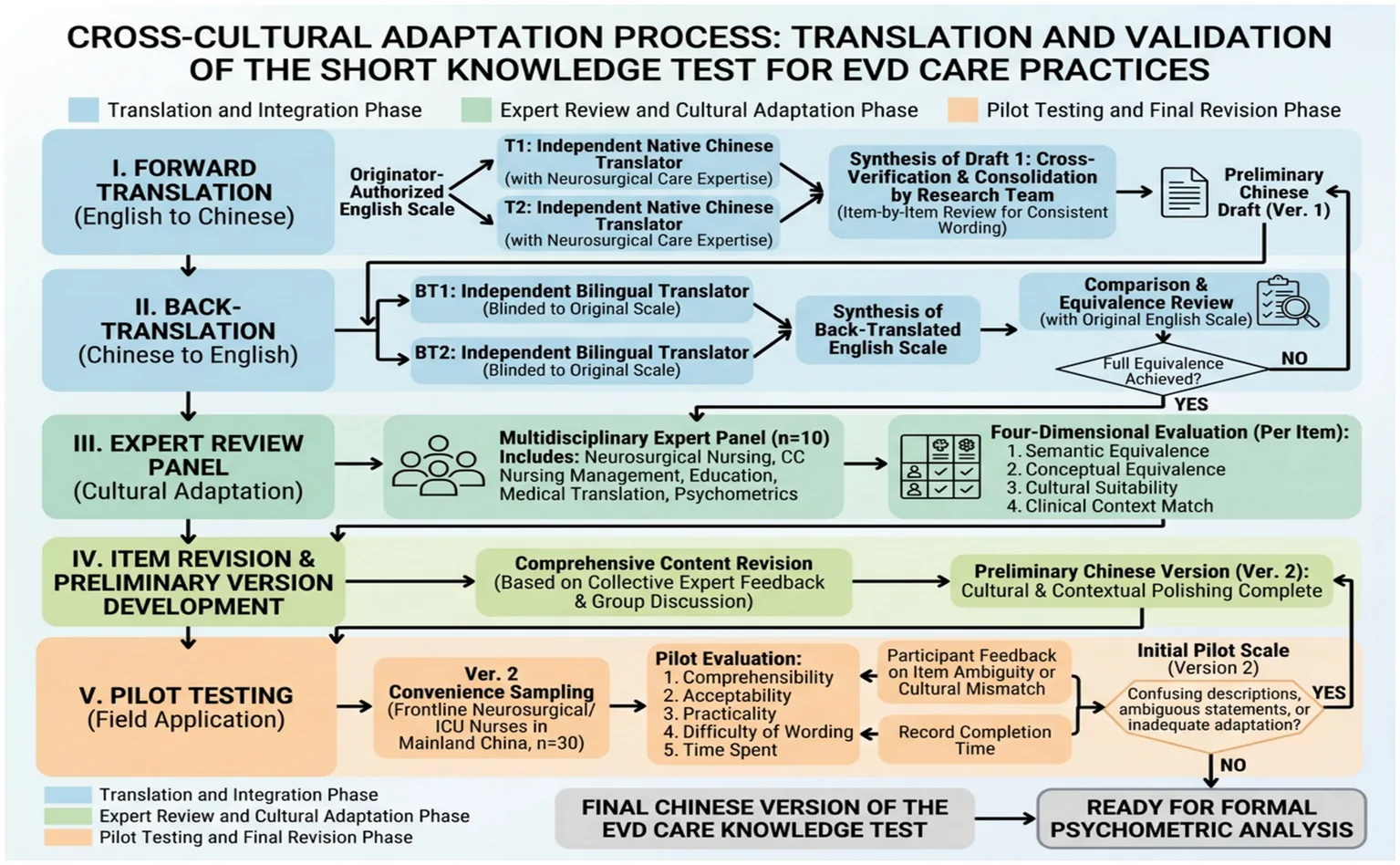
Flowchart of the translation and cross-cultural adaptation process of the English version of the instrument based on the Brislin translation model.

#### Translation and synthesis stage (forward translation and back-translation)

##### Step 1: Forward translation (English to Chinese)

After obtaining permission from the original instrument developer, two independent professional translators (T1 and T2), both native Chinese speakers with backgrounds in neurosurgical specialty nursing, independently translated the original English version of the instrument into Chinese. The two translators worked independently and were blinded to each other’s translations, resulting in two separate initial Chinese translations.

The research team compared and cross-checked the two translations item by item, focusing on the consistency of item wording and professional terminology. Discrepancies between the two translations were discussed collectively by the research team to reach a consensus on the wording and logical structure of the items. The two translations were then synthesized into a preliminary Chinese version (Version 1).

##### Step 2: Back-translation (Chinese to English)

Two independent bilingual translators (BT1 and BT2), who were proficient in both Chinese and English and had no access to the original English version of the instrument, independently back-translated the preliminary Chinese version (Version 1) into English under blinded conditions. Two separate back-translated English versions were thus obtained.

The research team synthesized the two back-translated versions and compared the synthesized English version item by item with the original English instrument to assess semantic and conceptual equivalence. Particular attention was paid to whether the core concepts, professional meanings, and logical structure of each item were consistent with those of the original instrument. When semantic discrepancies or conceptual deviations were identified, the research team revisited and revised the corresponding Chinese wording. This process of iterative comparison and revision continued until satisfactory equivalence between the back-translated and original versions was achieved before proceeding to the expert review stage.

#### Expert review and cross-cultural adaptation

A 10-member expert review panel was recruited via purposive sampling, covering neurosurgical specialized nursing, critical care nursing, nursing management, nursing education, clinical nursing, and psychometrics. Four experts had associate senior titles and six had senior titles; seven were master’s degree holders and three held doctoral degrees, with work experience of 21–33 years. The panel conducted systematic cultural adaptation and item evaluation for the equivalent qualified Chinese preliminary scale. Each item was assessed individually across four dimensions.

1. Semantic equivalence: Whether the wording was clear, fluent, and free of ambiguity;
2. Conceptual equivalence: Whether the core meaning of each item was consistent with the construct and measurement objective of the original instrument;
3. Cultural relevance: Whether the wording and question format were appropriate for the clinical nursing context in China and free from culture-specific expressions that might be inappropriate for Chinese healthcare settings;
4. Clinical contextual relevance: Whether the knowledge assessed by each item was consistent with the *Expert Consensus on External Ventricular Drainage Care* and routine clinical nursing practices in China.

All expert feedback was systematically summarized and discussed collectively by the research team. Items containing awkward or overly literal wording, culture-specific expressions originating from overseas healthcare settings, or question formats that were inconsistent with clinical nursing practices in China were revised and refined. Based on the experts’ feedback, several items underwent minor linguistic and semantic refinements to accommodate the linguistic expression habits and clinical context of Chinese nurses. Importantly, these modifications were made while retaining the original conceptual meaning and intended content of the instrument. The adaptations mainly involved terminology refinement, simplification of complex expressions, and contextual clarification, rather than substantive modification of the underlying construct.

The following six items were slightly modified during the cross-cultural adaptation process:

“Cerebrospinal fluid samples should be delivered to the laboratory without unnecessary delay, unless urgent patient care takes priority.” was rephrased as “Cerebrospinal fluid specimens should be promptly delivered to the laboratory for testing, unless urgent patient care takes priority,” “Cerebrospinal fluid samples should be obtained from a designated sampling port of the EVD system according to institutional protocol.” was rephrased as “Cerebrospinal fluid specimens should be collected from the designated sampling interface of the EVD system in accordance with relevant hospital protocols,” “The EVD drainage bag should be replaced in accordance with institutional protocols and before it becomes completely full” was rephrased as “The EVD drainage bag should be replaced in accordance with relevant hospital protocols and before it becomes completely full,” “The EVD system should be leveled according to a defined anatomical reference point specified in institutional protocols.” was rephrased as “The EVD system should be leveled according to the anatomical reference point specified in relevant hospital protocols,” “The EVD system should be maintained in an appropriate position to ensure safe drainage and to minimize contamination risk.” was rephrased as “The EVD system should be maintained in an appropriate position to ensure safe drainage and reduce the risk of contamination,” and “EVD lines should be clearly identified to prevent confusion with intravenous lines,” was rephrased as “EVD lines should be clearly labeled to prevent confusion with intravenous infusion lines”.

The modifications primarily reflected differences in terminology and linguistic expression rather than changes in item meaning. Specifically, “samples” was changed to “specimens” to use more appropriate clinical terminology; “without unnecessary delay” was simplified to “promptly” to improve conciseness and naturalness; and “sampling port” was changed to “sampling interface” to better align with the terminology used in the Chinese clinical context. The phrase “institutional protocols” was consistently adapted to “relevant hospital protocols” to improve contextual specificity and consistency with common organizational terminology. In addition, “a defined anatomical reference point specified in institutional protocols” was simplified to “the anatomical reference point specified in relevant hospital protocols,” while “minimize contamination risk” was revised to “reduce the risk of contamination” for greater linguistic naturalness. Finally, “identified” was changed to “labeled,” and “intravenous lines” was specified as “intravenous infusion lines” to enhance clarity and reduce potential ambiguity.

Overall, these changes were considered minor linguistic and semantic adaptations and did not alter the underlying construct, clinical meaning, or intended interpretation of the original items. The final Chinese version therefore maintained conceptual equivalence with the original instrument while improving linguistic clarity, cultural appropriateness, and clinical applicability among Chinese nurses. Following this comprehensive revision process, a culturally adapted preliminary Chinese version (Version 2) was developed.

#### Pilot testing and final revision

To evaluate whether the translated items were understandable and interpreted appropriately by nurses in the target population, pretesting (pilot testing) was conducted with a convenience sample of 30 nurses who met the predefined inclusion criteria. The second preliminary Chinese version of the instrument (Version 2) was administered during this small-scale field pilot study.

The pretesting focused on five aspects: (1) item comprehensibility; (2) acceptability of the response process; (3) clinical applicability; (4) difficulty of understanding the item wording; and (5) the time required to complete the entire instrument. The completion time for each participant was recorded. Participants were also invited to provide feedback on how they understood the items and to identify ambiguous or unclear wording, potentially confusing expressions, or any aspects of the items that appeared culturally inappropriate or inconsistent with the Chinese clinical context.

All feedback from the pilot testing was systematically reviewed by the research team. When participants identified unclear wording, ambiguity, or insufficient cultural adaptation, the corresponding items were returned to the revision process and further refined. When no substantial concerns regarding item comprehension, wording, or cultural applicability were identified, the revised version was finalized as the C-SKT-EVD. The finalized instrument was subsequently used for the large-scale psychometric evaluation.

This pilot testing was conducted as pretesting of item comprehension and interpretation rather than as a formal cognitive interviewing procedure; structured think-aloud techniques and formal cognitive probing were not used. Therefore, the pilot findings were used to assess the clarity and contextual appropriateness of the translated items, rather than to provide a formal cognitive-process evaluation of respondents’ interpretation.

### Study design and participants

This study adopted a cross-sectional psychometric validation design and was conducted among clinical nurses at the First Affiliated Hospital of Jinzhou Medical University, Liaoning Province, mainland China, from July 2026 to August 2026.

Inclusion Criteria: ① Were registered nurses holding a valid nursing license issued by the People’s Republic of China and currently practicing in mainland China; ② Were aged ≥18 years; ③ Had at least 6 months of clinical nursing experience and were currently engaged in clinical nursing practice; ④ Were currently working in neurosurgery, neurocritical care units, general intensive care units, emergency departments, general surgery, or other clinical departments that may admit patients requiring external ventricular drainage (EVD); ⑤ Were able to understand the study information and independently complete the questionnaire; and ⑥ Voluntarily participated in the study and provided informed consent. Exclusion Criteria: ① Were primarily engaged in non-clinical nursing roles, such as nursing management, education, research, or administrative work; ② Were nursing interns, visiting nurses, nursing assistants, or other individuals without registered nurse licensure.

### Sample size estimation

The sample size for the Rasch analysis was determined based on the desired precision of item calibration and the stability of parameter estimates. According to Linacre (18), the sample size required for Rasch measurement depends primarily on the desired precision of item calibration and the degree of person–item targeting, rather than solely on the number of items. For polytomous items, relatively small samples may provide stable item calibrations when the sample is well targeted to the items, whereas larger samples generally yield greater precision and stability of item parameter estimates. According to Linacre’s guidelines, a sample of approximately 150 participants is generally sufficient for obtaining stable item calibrations within ±0.5 logits at a 99% confidence level under favorable targeting conditions.

In the present study, the instrument comprised seven items designed to assess a unidimensional construct, with each item scored using a five-point response scale. Considering the desired measurement precision and the need for stable estimation of item and person parameters, a sample size of approximately 200 or more participants was considered adequate for the planned Rasch analysis. Ultimately, 242 participants were included in the study, providing sufficient response data for Rasch analysis and supporting the stability and precision of the resulting parameter estimates.

### Study instruments

Sociodemographic and Professional Characteristics Questionnaire: A self-developed questionnaire was used to collect participants’ demographic and professional characteristics, including gender, age, educational background, working years, professional title, and department.

Chinese Version of the Short Knowledge Test to Evaluate Awareness of External Ventricular Drain Care Practices (C-SKT-EVD): The C-SKT-EVD is a unidimensional, seven-item instrument comprising factually oriented statements describing recommended EVD care practices. Each item is rated on a 5-point Likert-type scale from 1 (strongly disagree) to 5 (strongly agree), with no reverse-scored items. All items represent correct statements regarding EVD care practices; therefore, higher total scores reflect greater endorsement of these statements and a higher level of self-reported knowledge of recommended EVD care practices (Supplementary material 1). Although “awareness” is retained in the original instrument title, the present study interprets the measured construct as self-reported knowledge of recommended EVD care practices. Accordingly, the scores are not interpreted as objectively tested factual knowledge or clinical competence.

### Data collection

Data were collected through an online questionnaire administered via Wenjuanxing, a widely used online survey platform in China. Before completing the survey, participants were provided with an introduction describing the purpose and significance of the study, the survey procedures, and the anonymous nature of participation. Participants were informed that all collected data would be used solely for research purposes and that participation was voluntary. Those who agreed to participate clicked the confirmation button, thereby providing electronic informed consent, and were then directed to complete the questionnaire. Participants who declined to participate could exit the survey at any time without providing any information. The questionnaire took approximately 1–3 min to complete.

To ensure data quality, all submitted questionnaires were reviewed by the research team. Responses were excluded if participants selected the same response option for most or all items or if the questionnaire was completed in less than 60 s, as these patterns were considered indicative of insufficient attention or engagement. A total of 280 questionnaires were distributed, of which 242 valid responses were obtained, yielding a valid response rate of 86.43%.

### Statistical analysis

Statistical analyses were performed using IBM SPSS 26.0, Winsteps version 3.72.3 and Python 3.13.15. Continuous variables were summarized as means and standard deviations (SDs) for normally distributed data, whereas non-normally distributed data were presented as medians and interquartile ranges (IQRs). Categorical variables were summarized as frequencies and percentages.

### Model selection

Two widely-used polytomous Rasch models, the Rating Scale Model (RSM) and the Partial Credit Model (PCM), were fitted for model comparison. The RSM constrains shared category thresholds across all items and is appropriate when all items have identical response categories with the same labels and meaning. By contrast, the PCM allows item-specific category thresholds without imposing equality constraints on category intervals. Since the RSM is a nested constrained version of the PCM, the likelihood-ratio test was applied for model comparison, together with the Akaike Information Criterion (AIC) and Bayesian Information Criterion (BIC). Lower values of AIC and BIC indicate better trade-off between model fit and parsimony. If the likelihood-ratio test was non-significant and yielded smaller information criterion values, the more parsimonious RSM would be adopted; otherwise, the PCM would be selected. After determining the optimal model, subsequent analyses including unidimensionality check, item fit, item-person distribution, reliability and DIF were performed.

### Normality test

The distributional characteristics of the total score and individual item scores of the instrument were visually assessed using Q–Q (quantile–quantile) plots (19). Data were considered approximately normally distributed when the data points were generally and evenly distributed along the diagonal reference line. When the data points showed substantial deviations from the diagonal reference line, the data were considered non-normally distributed. All descriptive statistical analyses and Q–Q plots were generated using SPSS version 26.0.

### Content validity and expert authority assessment

Content validity was evaluated by a multidisciplinary expert panel comprising 10 experts with expertise in neurosurgical nursing, critical care nursing, nursing management, nursing education, clinical nursing, and psychometrics. The experts were selected based on their professional expertise and clinical or academic experience relevant to EVD care and nursing measurement. Each expert independently evaluated the relevance of each item to the construct being measured using a 4-point Likert scale (1 = not relevant, 2 = somewhat relevant, 3 = quite relevant, and 4 = highly relevant). The item-level content validity index (I-CVI) was calculated as the proportion of experts assigning a rating of 3 or 4 to each item. The scale-level content validity index based on the average method (S-CVI/Ave) was calculated as the mean of the I-CVI values across all items. An I-CVI ≥ 0.78 and an S-CVI/Ave ≥ 0.90 were considered indicative of satisfactory content validity (20).

The authority of the expert panel was assessed using the expert authority coefficient (*Cr*), which was calculated as the mean of the experts’ judgment basis coefficient (*Ca*) and familiarity coefficient (*Cs*): $\mathrm{Cr}=\frac{\left(\mathrm{Ca}+\mathrm{Cs}\right)}{2}$ . The familiarity coefficient (*Cs*) reflected the experts’ self-rated familiarity with the relevant content, whereas the judgment basis coefficient (*Ca*) reflected the extent to which their judgments were based on theoretical analysis, practical experience, peer consultation, literature review, and other sources of evidence. A higher *Cr* value indicated a higher level of expert authority. In general, a *Cr* ≥ 0.70 was considered acceptable, whereas a *Cr* ≥ 0.80 indicated a high level of expert authority (21).

### Unidimensionality and local independence assessment

Unidimensionality was assessed using principal component analysis of standardized residuals (PCAR) to examine whether the items primarily measured a single underlying latent construct. In this study, the latent construct was defined as self-reported knowledge of recommended EVD care practices, reflected through graded endorsement of factually oriented care statements. Two primary indicators were examined: the proportion of raw variance explained by the Rasch measures and the eigenvalue of the first contrast in the standardized residuals. A variance explained by the Rasch measures of ≥50% and a first residual contrast eigenvalue of <2.0 were used as practical indicators of an acceptable predominantly unidimensional structure (22). These criteria were not considered definitive evidence of strict unidimensionality, particularly given the small number of items. Local independence was evaluated by examining correlations between standardized residuals for each pair of items after controlling for the Rasch latent trait. Residual correlations >0.30 were considered indicative of potential local dependence (23). This criterion was used to identify item pairs warranting further examination rather than to establish absolute independence. Because several items address clinically interconnected aspects of EVD management, residual associations were interpreted in conjunction with the conceptual content of the items.

### Item fit assessment

Item fit to the Rasch model was evaluated using the infit mean square (Infit MNSQ) and outfit mean square (Outfit MNSQ) statistics. According to the criteria proposed by Linacre, Infit MNSQ and Outfit MNSQ values within the range of 0.5–1.5 were considered indicative of acceptable model fit (24). Items exceeding this range were not automatically removed based solely on a single fit statistic; instead, decisions regarding item retention were made by considering the item content, theoretical relevance, and other psychometric properties. In addition, the point-measure correlation coefficient (PT-MEASURE CORR.) was used to evaluate the consistency between item responses and the estimated latent trait of respondents. A positive PT-MEASURE CORR. value indicates that item responses are aligned with changes in the underlying construct, whereas a negative value may suggest potential issues such as incorrect reverse scoring, item misinterpretation, or other response anomalies requiring further investigation. Items with relatively low PT-MEASURE CORR. values were further evaluated in conjunction with item fit statistics and content validity considerations (25).

### Assessment of rating scale category functioning

The functioning of the rating scale categories was examined based on several criteria. First, the observed frequency of responses in each category was evaluated. In principle, each category should have at least 10 observations to ensure stable estimation of category parameters. Second, the observed average measures were examined to determine whether they increased monotonically across ordered response categories, indicating an appropriate correspondence between respondents’ latent trait levels and rating categories. Third, the Infit MNSQ and Outfit MNSQ statistics for each category were assessed, with values between 0.5 and 1.5 considered acceptable, to determine whether the response patterns of each category were consistent with Rasch model expectations (24). Furthermore, the ordering of category threshold parameters (step calibration/structure calibration) was examined. Ideally, the thresholds should demonstrate a monotonic increasing order with reasonable distances between adjacent thresholds, indicating that respondents could distinguish between successive response categories appropriately (23). Finally, category probability curves were inspected to evaluate the dominant regions and peak distributions of each response category along the latent trait continuum. A well-functioning rating scale should demonstrate distinct and mutually exclusive regions of dominance for each category. These analyses were combined to comprehensively evaluate the appropriateness of the five-category response structure (26).

### Item–person map (wright map)

An item–person distribution map (item–person map/Wright map) was generated to visually display respondents’ latent trait levels and item difficulty parameters on the same logit scale (27). This analysis was performed to evaluate the correspondence between the distribution of item difficulties and the distribution of respondents’ latent traits, thereby assessing the measurement targeting and coverage of the scale across different levels of the underlying construct. By comparing the person ability distribution with the item difficulty distribution, potential gaps in item coverage were identified. A substantial concentration of respondents’ trait levels at the upper or lower end of the item difficulty continuum may indicate potential ceiling or floor effects, respectively (28). Conversely, large gaps between the distributions of person abilities and item difficulties suggest that the scale may have limited measurement precision for respondents at corresponding levels of the latent trait.

### Measurement precision analysis

The Test Information Function (TIF) was used to evaluate the measurement precision of the scale across different levels of the latent trait. The TIF reflects the amount of information provided by the scale at different levels of respondent ability (Measure). Higher information values indicate greater measurement precision and smaller standard errors of measurement at the corresponding ability levels. The TIF curve was plotted to examine the measurement precision and applicable range of the scale across different levels of EVD nursing knowledge (29).

### Separation index and separation reliability

Item separation index, item separation reliability, person separation index, and person separation reliability were calculated to evaluate the ability of the scale to distinguish among different levels of item difficulty and respondent ability. Based on previous studies, a separation index of ≥1.50 indicates acceptable separation capability, ≥2.00 indicates good separation capability, and ≥3.00 indicates excellent separation capability. A separation reliability value of ≥0.70 was considered indicative of satisfactory discriminative ability (30).

### Differential item functioning (DIF) analysis

DIF was examined across gender, professional title, years of work experience, and department. For each grouping variable, item functioning was compared after controlling for the overall level of the latent trait. Welch’s *t*-test and MH were treated as complementary statistical indicators rather than independent requirements that must both be significant (31).

Both statistical significance and the magnitude of the DIF Contrast were considered when evaluating potential DIF. A DIF Contrast of 1.0 logits was prespecified as the criterion for substantial DIF (32). Statistical significance alone was not considered sufficient to classify an item as exhibiting meaningful DIF, because statistically detectable differences may occur with relatively small effect sizes. Accordingly, an item was considered to show substantial DIF only when the magnitude of the DIF Contrast reached or exceeded 1.0 logits and was accompanied by statistical evidence from the DIF analyses. DIF findings that were statistically significant but had a DIF Contrast below 1.0 logits were interpreted as statistically detectable but practically small differences rather than substantial DIF. For grouping variables with more than two categories, pairwise comparisons were conducted to identify specific category pairs contributing to potential DIF. This approach was used to locate subgroup-specific differences rather than treating the overall multi-category variable as a single homogeneous comparison. Because multiple item-by-group and pairwise comparisons were performed, individual *p* values were interpreted cautiously and were not considered sufficient evidence of meaningful DIF in isolation. The overall interpretation therefore emphasized the consistency between statistical evidence and DIF Contrast magnitude rather than statistical significance alone. The absence of statistically significant DIF was not interpreted as evidence of complete measurement invariance. Instead, the analysis was used to determine whether practically substantial item-level DIF could be identified within the examined subgroups (33, 34).

## Results

### Model selection

Model comparison between the RSM and PCM was conducted. The log-likelihood of the RSM was −2,375.79 with 11 parameters (AIC = 4,773.57, BIC = 4,811.95), while the PCM yielded a log-likelihood of −2,366.25 with 28 parameters (AIC = 4,788.50, BIC = 4,886.19). Although the PCM achieved a slightly better log-likelihood, it required more parameters. The likelihood ratio test was non-significant (*χ*^2^ = 19.07, *df* = 17, *p* = 0.3245), indicating no statistically significant difference in model fit between the two models. Both AIC and BIC favoured the RSM, with an Akaike weight of 0.9994 and BIC weight close to 1.0000. Considering model parsimony and overall fit, the RSM was selected for subsequent Rasch analyses (Table 1).

**Table 1:** Comparison of model fit indices and likelihood ratio test (LRT) results.

Comparison of model fit indices			
Metric	RSM	PCM	Preferred model
Log-likelihood	−2,375.79	−2,366.25	PCM (better fit)
Number of parameters (k)	11	28	RSM (more parsimonious)
AIC	4,773.57	4,788.5	RSM
BIC	4,811.95	4,886.19	RSM
Akaike weight	0.9994	0.0006	RSM
Schwarz (BIC) weight	≈1.0000	≈0.0000	RSM

Likelihood ratio test (LRT)			
*χ*^2^ statistic	Degrees of freedom (*df*)	*p*-value	Significance (*α* = 0.05)
19.07	17	0.3245	Non-significant

### Translation and cross-cultural debugging

#### Assessment of normality and score distribution

Normality of the total score and individual item scores of the C-SKT-EVD was assessed using Q–Q normal probability plots. The results showed that the observed data points for both the total scale score and all individual items closely aligned with the theoretical diagonal line, indicating that the total score and item scores approximately followed a normal distribution (Figure 2). In the present study, the mean total score of the scale was 21.18 ± 6.619. The mean scores for the seven items were 3.95 ± 1.292, 3.60 ± 1.388, 3.18 ± 1.408, 3.04 ± 1.459, 2.68 ± 1.412, 2.48 ± 1.367, and 2.26 ± 1.298, respectively.

**Figure 2:**
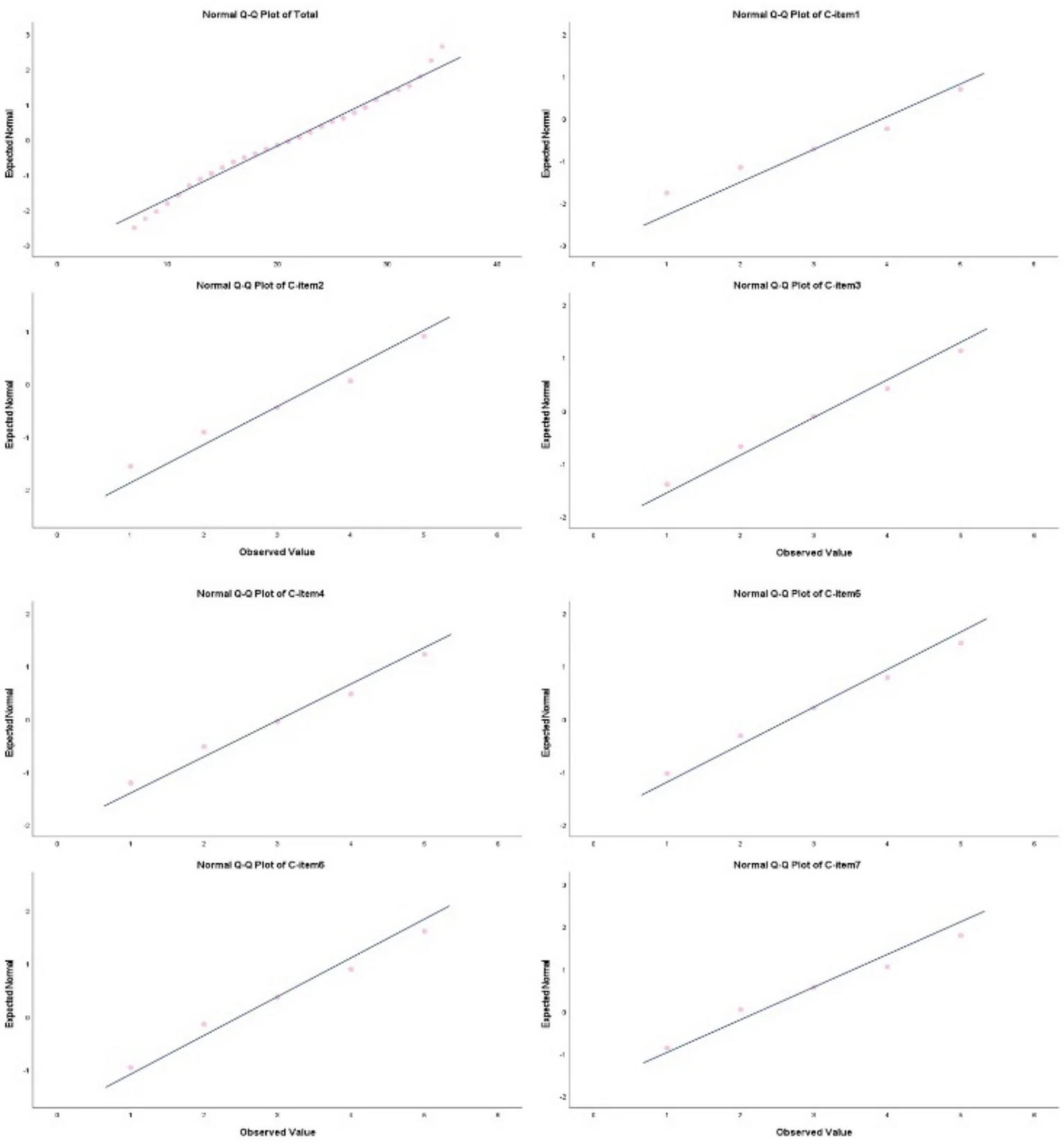
Normal Q-Q plots for the total score and each item of the C-SKT-EVD. The pink dots represent observed quantiles, and the blue solid line denotes the theoretical normal reference line. Points closely following the reference line indicate approximate normal distribution of the data.

#### Content validity and expert authority assessment

The content validity of the C-SKT-EVD was evaluated by calculating the item-level content validity index (I-CVI) and scale-level content validity index (S-CVI). The results demonstrated that the I-CVI values of all items ranged from 0.80 to 1.00, and the S-CVI was 0.95, indicating satisfactory content validity of the scale. In addition, the expert authority coefficient was assessed based on the coefficients of familiarity (*Cs*) and judgment basis (*Ca*). The results showed that *Cs*, *Ca*, and *Cr* were 0.93, 0.94, and 0.935 (Tables 2, 3), respectively, suggesting a high level of expert authority in the content evaluation process.


$$\text{The expert judgment basis}\;\mathrm{Ca}=\frac{\begin{array}{l} (0.5*7+0.4*2+0.3*1)+\\ (0.3*7+0.2*3)+\\ (0.1*6+0.1*2+0.1*2)+\\ \left(0.1*1+0.1*2+0.1*7\right) \end{array}}{10}=0.93.$$

$$\text{The expert familiarity}\;\mathrm{Cs}=\frac{8*1+0.8*1+0.6*1}{10}=0.94$$

**Table 2:** Assignment of expert judgment basis (*n* = 10).

Judgment basis (Ca)	Degree classification of influences on expert judgment		
	High	Medium	Low
Practical experience	0.5 (7)	0.4 (2)	0.3 (1)
Theoretical analysis	0.3 (7)	0.2 (3)	0.1 (0)
Reference to domestic and foreign literature	0.1 (6)	0.1 (2)	0.1 (2)
Subjective perception	0.1 (1)	0.1 (2)	0.1 (7)

The figures enclosed in parentheses stand for the quantity of participating experts.

**Table 3:** Assignment of expert familiarity (*n* = 10).

Familiarity level (Cs)	Very familiar	Relatively familiar	Moderately familiar	Slightly familiar	Unfamiliar
Expert self-assessment	1 (8)	0.8 (1)	0.6 (1)	0.4 (0)	0.2 (0)

The figures enclosed in parentheses stand for the quantity of participating experts.

#### Basic characteristics of the study participants

A total of 242 nurses were included in this study. Among them, 69.0% were female and 31.0% were male. The majority of participants were aged 20–39 years, accounting for 75.2% of the total sample. Most participants had a bachelor’s degree (59.9%). Nearly half of the nurses had 1–5 Working years (45.0%). Regarding professional titles, nurses and nurse practitioners constituted the majority, accounting for 75.2% collectively. The participants were mainly recruited from clinical departments closely associated with EVD care, such as neurosurgery departments and NICU (Table 4).

**Table 4:** Demographic characteristics of participants (*n* = 242).

Demographic variables		Frequency (*n*)	Percentage (%)
Gender	Female	167	69.0
	Male	75	31.0
Age (years)	20–29	97	40.1
	30–39	85	35.1
	40–49	43	17.8
	50–59	15	6.2
Educational background	Junior college	73	30.2
	Bachelor degree	145	59.9
	Postgraduate and above	24	9.9
Working years	1–5	109	45.0
	6–10	85	35.1
	>10	48	19.8
Professional title	Nurse	97	40.1
	Senior Nurse	85	35.1
	Nurse-in-charge	48	19.8
	Associate Chief Nurse and above	12	5.0
Department	Neurosurgery	97	40.1
	NICU	61	25.2
	ICU	48	19.8
	Emergency	36	14.9

NICU, Neurosurgery Intensive Care Unit; ICU, Intensive care units.

#### Unidimensionality assessment

Unidimensionality of the C-SKT-EVD was evaluated using standardized residual variance analysis and a scree plot. The raw variance explained by the Rasch measures was 55.1%, while the model-explained variance was 55.9%, both exceeding the prespecified 50% criterion. The eigenvalue of the first contrast in the standardized residuals was 1.4, which was below the prespecified threshold of 2.0. The eigenvalues of the second to fifth residual contrasts were 1.2, 1.2, 1.1, and 1.1, respectively, showing a gradual and relatively flat decline (Table 5).

**Table 5:** Results of the unidimensionality assessment of the C-SKT-EVD.

Source of variance	Eigenvalue	Empirical (%)	Modeled (%)
Total raw variance in observations	15.6	100.0	100.0
Raw variance explained by measures	8.6	55.1	55.9
Raw variance explained by persons	3.6	22.9	23.2
Raw variance explained by items	5.0	32.2	32.7
Raw unexplained variance (total)	7.0	44.9	44.1
Unexplained variance in 1st contrast	1.4	8.7	19.4
Unexplained variance in 2nd contrast	1.2	7.9	17.7
Unexplained variance in 3rd contrast	1.2	7.5	16.7
Unexplained variance in 4th contrast	1.1	7.1	15.9
Unexplained variance in 5th contrast	1.1	6.9	15.3

The scree plot of residual variances did not show a prominent secondary peak or a clear steep inflection point (Figure 3). No prominent secondary dimension was therefore detected. However, given the small number of items, the sensitivity of PCAR to detect weak secondary dimensions is limited. In addition, several items address clinically interconnected aspects of EVD management, which may contribute to residual associations without necessarily representing distinct latent dimensions. Taken together, the findings are consistent with a predominantly unidimensional structure, with the seven items primarily reflecting a common latent continuum of self-reported knowledge of recommended EVD care practices.

**Figure 3:**
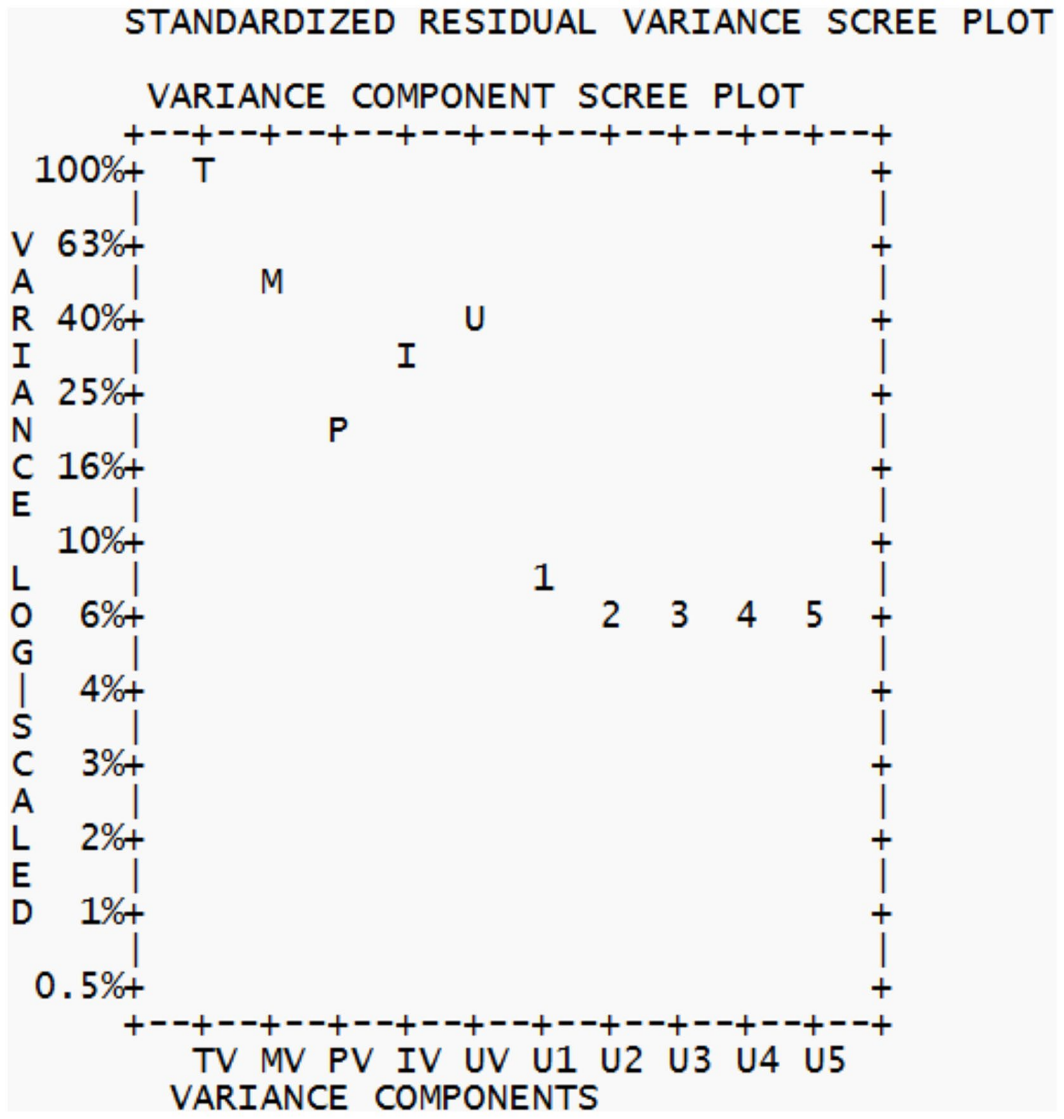
Principal component analysis of standardized Rasch residuals (PCAR) for the C-SKT-EVD. The vertical axis represents the log-scaled proportion of standardized residual variance, and the horizontal axis represents the residual variance components. T, M, P, I, and U denote the total, measure, person, item, and unexplained variance components, respectively. Components 1–5 represent the first five residual contrasts extracted from the standardized Rasch residuals.

#### Local independence

The assumption of local independence was examined by assessing the correlations among standardized residuals. The largest residual correlation between item pairs was −0.15, and all residual correlations ranged from −0.25 to −0.15, which were below the recommended threshold of 0.30. These findings indicated that no substantial local dependence existed among the seven items, supporting the local independence assumption of the Rasch model.

#### Item fit statistics

Item fit statistics of the C-SKT-EVD were evaluated using the Rasch model. The item difficulty measures ranged from −0.97 to 0.79 logits, with a mean item difficulty of 0.00 logits, indicating an appropriate spread of item difficulty and a good match between item difficulty and participants’ ability levels. All items demonstrated acceptable fit statistics, with Infit MNSQ values ranging from 0.94 to 1.16 and Outfit MNSQ values ranging from 0.94 to 1.07, all within the recommended range of 0.5–1.5. The corresponding Infit ZSTD values ranged from −0.7 to 1.6, and Outfit ZSTD values ranged from −0.6 to 0.7, all falling within the acceptable criterion of −2 to +2, indicating no significant item misfit. The PT-Measure Corr. ranged from 0.63 to 0.70, demonstrating good associations between individual items and the underlying latent trait measured by the scale. Furthermore, the observed average exact-match rate was 39.9%, which was highly consistent with the expected value (39.6%), suggesting satisfactory model-data fit and accurate prediction by the Rasch model (Table 6).

**Table 6:** Rasch model item fit statistics of the C-SKT-EVD.

Item	Measure	Model S.E.	Infit MNSQ	Infit ZSTD	Outfit MNSQ	Outfit ZSTD	PT-Measure Corr.	PT-Measure Exp.	Exact Match OBS%	Exact Match EXP%
C-item 7	0.79	0.07	0.99	0	0.98	−0.1	0.65	0.66	42.7	41
C-item 6	0.55	0.07	0.97	−0.3	0.95	−0.5	0.69	0.67	46	40
C-item 5	0.35	0.06	0.94	−0.7	0.96	−0.3	0.69	0.68	38.5	38.3
C-item 4	0	0.06	0.99	−0.1	0.94	−0.6	0.7	0.68	33.5	36.6
C-item 3	−0.15	0.06	1	0.1	1.03	0.3	0.67	0.68	38.9	35.7
C-item 2	−0.57	0.07	1.1	1.2	1.07	0.7	0.64	0.66	31.8	40.1
C-item 1	−0.97	0.07	1.16	1.6	1.07	0.6	0.63	0.64	47.7	45.3
Mean	–	0	0.07	1.02	0.2	1	0	–	–	39.9
S.D.	–	0.58	0	0.07	0.8	0.05	0.5	–	–	5.6

#### Category functioning analysis of the C-SKT-EVD

Category functioning of the C-SKT-EVD was evaluated to examine the appropriateness of the five-point rating scale structure. The results demonstrated that the average observed measures across the five response categories increased monotonically with increasing category levels, ranging from −1.06 to 1.21 logits. The step calibrations between adjacent categories were ordered as expected (−0.52, −0.38, 0.31, and 0.59 logits), indicating no disordering of category thresholds. The category fit statistics showed that the Infit and Outfit MNSQ values for all response categories ranged from 0.89 to 1.10, which were within the recommended range of 0.5–1.5, suggesting satisfactory category-model fit (Table 7). Furthermore, the category probability curves exhibited distinct and ordered peaks without substantial overlap or merging between adjacent categories, indicating that each response category effectively differentiated respondents with different levels of the latent trait (Figure 4). Overall, the five-category response structure of the C-SKT-EVD demonstrated appropriate functioning, and no category collapsing or modification was required.

**Table 7:** Results of the category functioning analysis of the C-SKT-EVD.

Category label	Observed count	Observed average measure	Structure calibration	Infit MNSQ	Outfit MNSQ
1	386	−1.06	None	1.05	1.08
2	285	−0.5	−0.52	1.04	0.99
3	321	−0.01	−0.38	0.96	0.89
4	303	0.48	0.31	1.1	1.02
5	399	1.21	0.59	0.96	0.98

**Figure 4:**
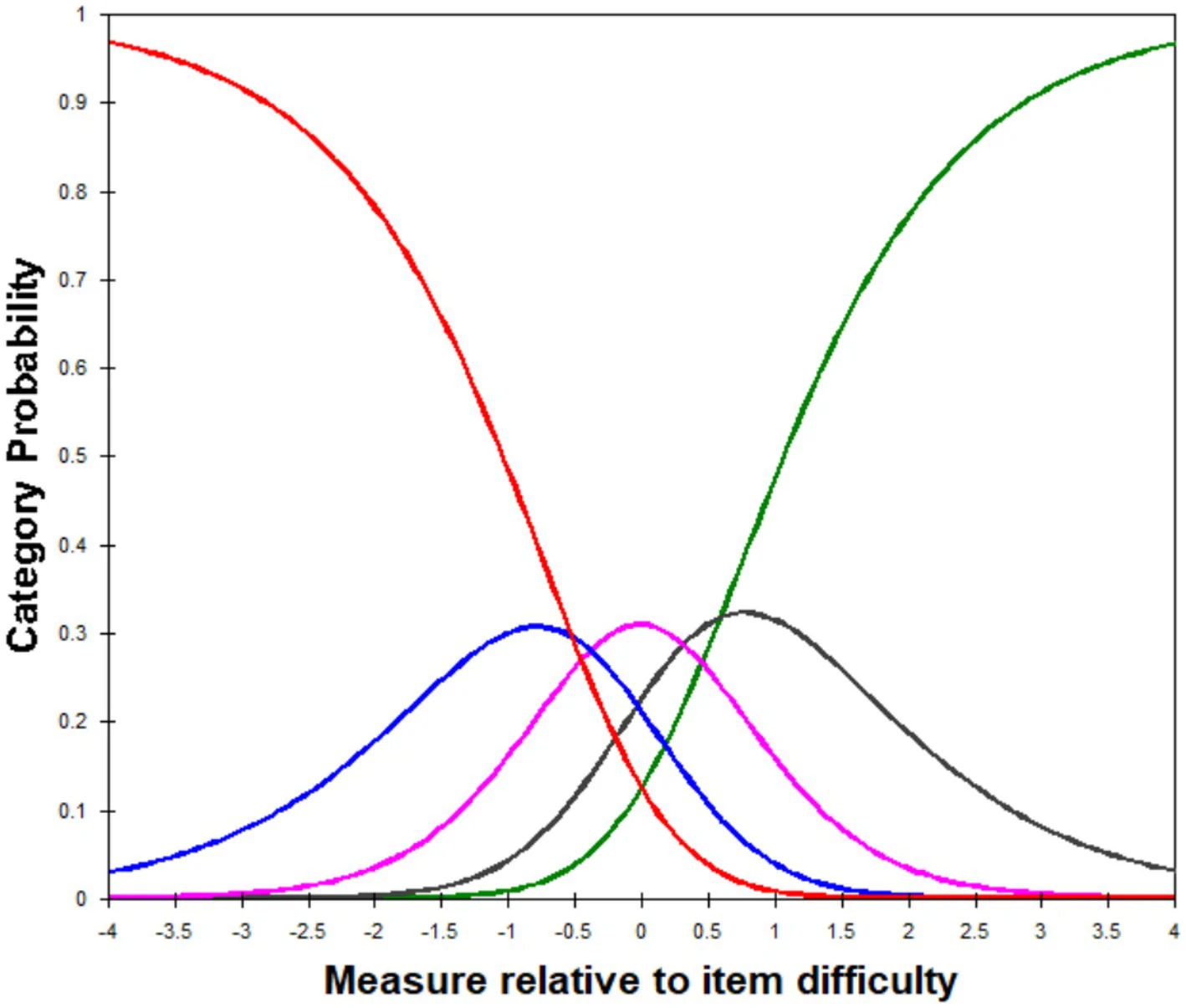
Category characteristic curves of the C-SKT-EVD for a 5-point Likert-type item based on the Rasch model. The red, blue, magenta, black and green curves denote the probability of selecting response category 1, 2, 3, 4 and 5, respectively. The *x*-axis is person ability (logit) relative to item difficulty; the *y*-axis is the probability of choosing a given response category. Intersections between adjacent curves indicate the threshold positions between consecutive response categories.

#### Person–item map

The Wright map was used to examine the targeting between item difficulty and participants’ ability. As shown in Figure 5, the seven items covered a difficulty range of −1.0 to +0.8 logits, whereas participants’ ability ranged from −2.5 to +2.8 logits. The mean person ability was approximately +0.3 logits, which was higher than the mean item difficulty (0 logits), suggesting that the scale was generally slightly easy for the sampled nurses. A ceiling effect was observed, with approximately 20–25% of participants demonstrating ability estimates above the difficulty of the most difficult item (C-item7), indicating limited capacity to discriminate among nurses with high EVD-care knowledge. The floor effect was mild, affecting approximately 7–10% of participants whose ability was below the easiest item (C-item1). Overall, the targeting between item difficulty and nurses’ ability was acceptable, although the shortage of items at the high ability end resulted in a degree of ceiling effect.

**Figure 5:**
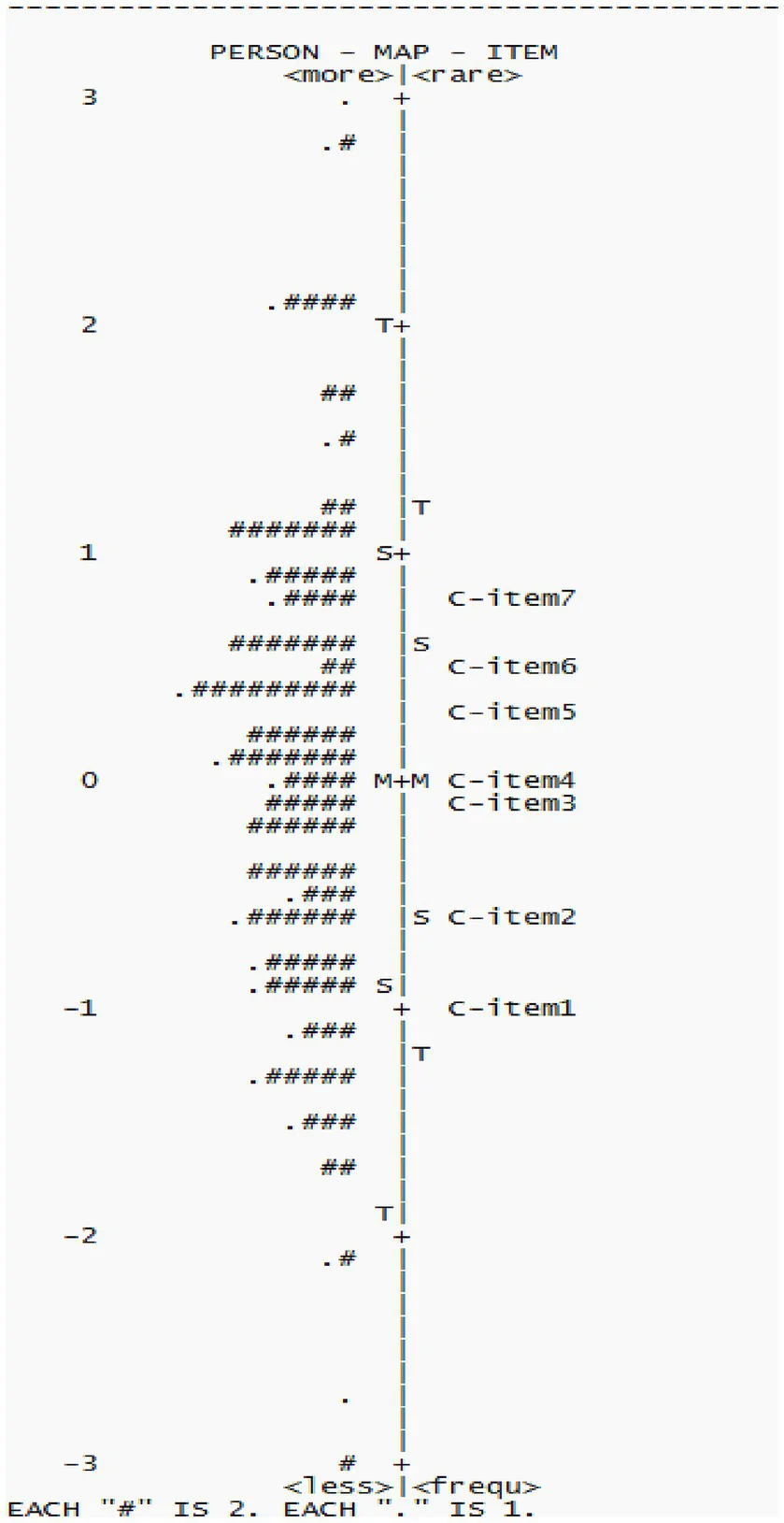
Person-item map of the C-SKT-EVD based on the Rasch model. The vertical axis is the logit scale. The left side shows the distribution of respondents’ ability, where “#” denotes 2 participants and “.” denotes 1 participant. The right side displays the difficulty locations of seven items (C-item1 to C-item7). The alignment between person ability and item difficulty reflects the match between sample and instrument.

#### Measurement precision

The TIF analysis showed that the TIF curve of the C-SKT-EVD exhibited a unimodal distribution, with a peak information value of approximately 8.5. The peak was located at approximately Measure = 0 logits, indicating that the scale provided the highest measurement precision for participants whose EVD nursing knowledge levels were close to the overall mean. Within the range of approximately −1 to +1 logits (Figure 6), the scale provided relatively abundant measurement information, whereas the amount of information gradually decreased among participants with extremely low or extremely high ability levels. These findings suggest that the C-SKT-EVD has relatively high measurement precision for individuals with moderate levels of EVD nursing knowledge.

**Figure 6:**
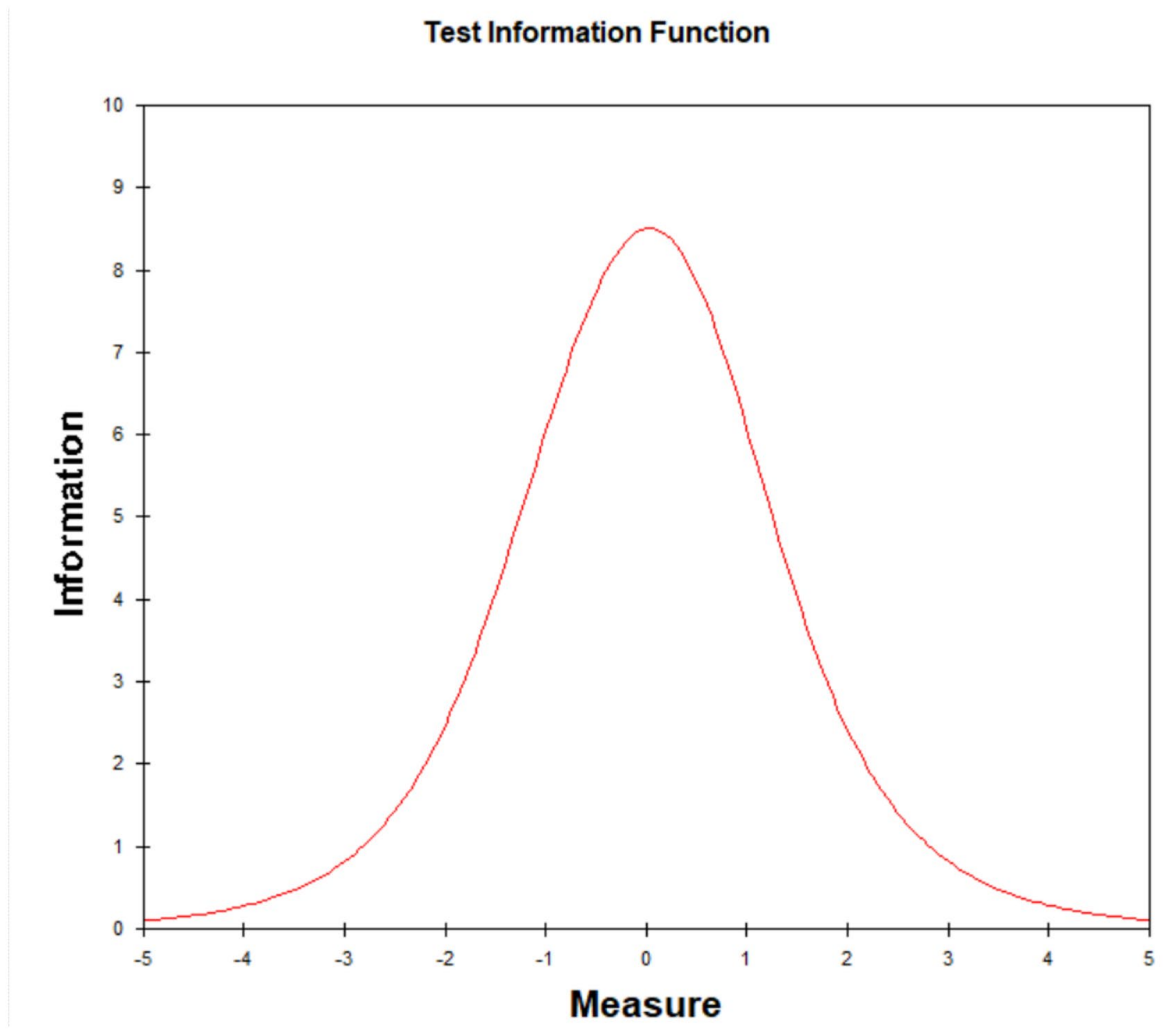
Test information function of the C-SKT-EVD based on the Rasch model. The *x*-axis denotes person ability in logit units; the *y*-axis represents test information. The peak of the curve indicates the ability range where the scale provides the highest measurement precision.

#### Separation reliability and separation indices

The person separation index was 1.86, with a Rasch person reliability of 0.78, indicating that the instrument provided moderate discrimination among respondents along the underlying self-reported knowledge continuum and could distinguish approximately two broad levels of the latent trait. This person reliability estimate should be interpreted as a Rasch measurement statistic reflecting the reproducibility of the relative ordering of respondents, rather than as an index of classical internal consistency such as Cronbach’s alpha.

The item separation index was 8.48, with an item reliability of 0.99, indicating that the ordering of item difficulties was estimated with high stability in the present sample. The high item separation and reliability support the stability of the item hierarchy, although they do not imply equivalent precision at the individual respondent level.

#### Differential item functioning analysis

DIF was examined across gender, professional title, years of work experience, and department using Welch’s *t*-test and the Mantel–Haenszel (MH) chi-square test, with both statistical significance and DIF Contrast magnitude considered in the interpretation. A DIF Contrast of 1.0 logits was prespecified as the criterion for substantial DIF.

Across gender groups, DIF Contrast values ranged from −0.18 to 0.31 logits. C-item4 showed a statistically significant difference in the Welch’s *t*-test (*t* = 2.18, *p* = 0.0307), but the DIF Contrast was only 0.31 logits, and the MH test was not statistically significant (*χ*^2^ = 4.082, *p* = 0.0501). Thus, the observed difference was considered statistically detectable but practically small.

Across professional title groups, the absolute DIF Contrast values ranged from 0.00 to 0.60 logits, with none exceeding the 1.0-logit criterion. No comparison was statistically significant in the Welch’s *t*-test (all *p* > 0.05). Although the MH test identified a statistically significant difference for C-item5 between professional title groups 2 and 4 (*χ*^2^ = 4.3100, *p* = 0.0379), the corresponding DIF Contrast was only 0.58 logits, and the Welch’s *t*-test was not statistically significant (*p* = 0.0859). Therefore, this finding was interpreted as a small, statistically detectable subgroup difference rather than substantial DIF.

Across years of work experience, the absolute DIF Contrast values ranged from 0.01 to 0.35 logits, with none exceeding 1.0 logits. C-item7 showed statistically significant differences between experience groups 1 and 2 in both the Welch’s *t*-test (*t* = 2.08, *p* = 0.0386) and the MH test (*χ*^2^ = 6.1579, *p* = 0.0131). However, the corresponding DIF Contrast was only 0.32 logits, indicating a small magnitude of difference.

Across departments, the absolute DIF Contrast values ranged from 0.01 to 0.59 logits, again remaining below the 1.0-logit criterion. C-item3 showed statistically significant differences in selected pairwise comparisons, including between department groups 1 and 2 in the Welch’s *t*-test (*t* = 2.05, *p* = 0.0429) and between groups 1 and 3 in the MH test (*χ*^2^ = 4.9773, *p* = 0.0257). C-item6 showed statistically significant differences between department groups 1 and 2 (DIF Contrast = −0.57 logits, *t* = −3.06, *p* = 0.0028; MH *χ*^2^ = 8.5506, *p* = 0.0035) and between groups 1 and 4 (DIF Contrast = −0.59 logits, *t* = −3.33, *p* = 0.0012; MH *χ*^2^ = 7.1185, *p* = 0.0076). Nevertheless, all observed DIF Contrasts remained below 1.0 logits.

Overall, several statistically significant differences were identified by the Welch’s *t*-test and/or MH test. However, none of the observed DIF Contrasts reached the prespecified threshold of 1.0 logits. Therefore, no practically substantial DIF was identified across gender, professional title, years of work experience, or department. The statistically significant findings were interpreted as relatively small subgroup differences rather than evidence of substantial item-level measurement bias. Detailed pairwise DIF results are provided in Supplementary material 2.

## Discussion

The present study represents an initial psychometric evaluation of the Chinese version of the Short Knowledge Test to Evaluate Awareness of External Ventricular Drain Care Practices (C-SKT-EVD) among Chinese nurses. Following cross-cultural adaptation, the instrument demonstrated generally acceptable measurement properties when examined using the Rasch Rating Scale Model. The findings supported the unidimensionality of the instrument, appropriate functioning of the response categories, satisfactory item fit, reasonable person–item targeting, acceptable person-level reliability, and stable estimation of the item hierarchy. Although several statistically significant subgroup differences were detected in the DIF analyses, none of the items demonstrated substantial DIF according to the predefined 1.0-logit criterion. Taken together, these findings provide preliminary evidence supporting the use of the C-SKT-EVD as a brief measure of nurses’ self-reported knowledge of recommended EVD care practices in the Chinese context. Importantly, the intended role of the instrument is knowledge screening and educational needs assessment rather than comprehensive clinical competency assessment. An important conceptual consideration is the distinction between objectively tested knowledge and self-reported knowledge. The C-SKT-EVD uses seven factually oriented statements describing recommended EVD care practices, but respondents indicate their degree of agreement on a five-point Likert-type scale rather than selecting objectively correct or incorrect answers. Therefore, the instrument should not be interpreted as a conventional objective knowledge test in which the number or proportion of correct answers is directly scored. Instead, its Rasch measures represent a graded continuum of self-reported knowledge and awareness reflected by respondents’ endorsement of recommended EVD care statements. Agreement with a correct statement may also be influenced by confidence, familiarity with institutional practice, perceived norms, or response tendencies. Accordingly, the present study retains the original construct of EVD care knowledge while explicitly limiting interpretation to self-reported knowledge and awareness rather than objectively verified factual knowledge or clinical competence.

The satisfactory content validity findings should be considered in relation to the cross-cultural adaptation process. Although the original instrument was developed to assess nurses’ knowledge of EVD care practices, direct linguistic translation alone may not ensure that clinical concepts are interpreted consistently across healthcare settings. During the adaptation process, terminology and expressions related to EVD equipment, drainage systems, leveling, sampling, and nursing procedures were reviewed and adjusted to improve their comprehensibility and clinical relevance for Chinese nurses. Expert evaluation and pilot testing further supported the clarity and relevance of the items. These procedures are particularly important for a clinical knowledge instrument because differences in terminology or local clinical conventions may introduce variation in responses that is unrelated to the underlying construct. The present content validity findings therefore provide an important foundation for subsequent psychometric evaluation. Nevertheless, content validity is inherently context-dependent. The seven items represent a focused set of recommended EVD care practices and should not be interpreted as providing comprehensive coverage of all knowledge required for EVD-related nursing care. Further evaluation in larger and more diverse Chinese nursing populations will be necessary to determine whether the current item content remains relevant across different clinical settings and nursing groups.

The Rasch findings provide additional evidence regarding the measurement properties of the seven-item instrument. Overall, the fit statistics indicated that the observed response patterns were broadly consistent with the expectations of the Rasch model, while the item–measure relationships were in the expected direction. Together with the evidence supporting unidimensionality, these findings suggest that the seven items contributed to the measurement of a common underlying construct. The absence of substantial local dependence further indicates that the information provided by individual items was not dominated by excessive redundancy. These findings are particularly relevant for a brief instrument because each item needs to contribute meaningful information to the construct being assessed. At the same time, the limited number of items means that the C-SKT-EVD should be regarded as a focused measure of knowledge of selected EVD care practices rather than an exhaustive assessment of all knowledge domains relevant to EVD care. The instrument’s brevity may enhance its feasibility for routine knowledge screening, but this advantage should be considered alongside its limited breadth of content coverage. The functioning of the five response categories supports retention of the original response structure in the Chinese version. The ordered category thresholds and increasing observed measures indicated that respondents with higher levels of the measured trait tended to select higher response categories. This pattern suggests that the response options were sufficiently differentiated to represent increasing levels of endorsement of the recommended EVD care practices. Nevertheless, appropriate category functioning should be interpreted as evidence that the response scale performed adequately in the present sample rather than as evidence that the five-category structure will function identically across all Chinese nursing populations. Differences in educational background, clinical experience, or familiarity with EVD care may influence how nurses use response categories (35). Future studies involving different nurse populations and clinical contexts should therefore continue to examine whether the response categories remain ordered and sufficiently discriminating. The person–item distribution provides a more nuanced interpretation of the measurement properties of the C-SKT-EVD. The item difficulties were concentrated primarily in the central portion of the logit scale, whereas participants’ person measures covered a somewhat wider range, particularly toward the higher end. The substantial overlap between person measures and item difficulties indicates that the instrument was reasonably targeted to the levels of self-reported knowledge represented in the present sample. However, the relatively limited coverage at the extreme ends of the latent continuum suggests that the instrument may provide less discrimination among nurses with exceptionally low or exceptionally high levels of self-reported knowledge. The test information function showed a similar pattern, with the greatest measurement information concentrated around the central portion of the latent trait and reduced information toward the lower and upper extremes. Thus, measurement precision was not uniform across the full continuum of self-reported knowledge. The DIF findings should be interpreted cautiously. Several statistically significant differences were identified by the Welch’s *t*-test and/or Mantel–Haenszel *χ*^2^ test across gender, professional title, years of work experience, and department. However, all corresponding DIF Contrast values remained below the prespecified threshold of 1.0 logits. Therefore, none of the items demonstrated practically substantial DIF according to the predefined criterion. The statistically significant findings should not be interpreted solely on the basis of *p* values, particularly given the multiple item-by-group and pairwise comparisons conducted in the DIF analyses. Small *p* values may reflect statistically detectable subgroup differences that are not necessarily large enough to have meaningful implications for measurement. In the present study, the relatively small DIF Contrasts associated with the statistically significant comparisons suggest that these differences were limited in magnitude. Some of the observed subgroup differences may reflect differences in clinical experiences, professional responsibilities, or working environments rather than substantial measurement bias. For example, nurses working in different departments or holding different professional titles may have different opportunities to encounter EVD-related procedures and clinical situations (14). Such contextual differences could influence responses to individual knowledge items without necessarily indicating problematic item functioning. For variables with more than two categories, pairwise comparisons were used to identify specific subgroup contrasts that might contribute to DIF. However, the results of these pairwise analyses should be interpreted cautiously because the number of statistical comparisons increases with the number of subgroup pairs. In addition, some subgroups may have had relatively small sample sizes, which may reduce statistical power to detect DIF. Accordingly, nonsignificant DIF tests should not be interpreted as evidence of complete measurement invariance Overall, the present findings indicate that no practically substantial item-level DIF was identified under the predefined criterion, rather than demonstrating complete measurement invariance across all examined subgroups. The relatively small DIF Contrasts observed for several statistically significant comparisons warrant further investigation in larger and more heterogeneous samples, particularly if the C-SKT-EVD is subsequently used to compare knowledge levels across different nursing populations.

An important implication of these findings concerns the distinction between self-reported knowledge assessment and actual clinical performance. EVD care is a specialized nursing activity that requires nurses not only to understand recommended practices but also to perform procedures accurately and consistently in clinical situations. The C-SKT-EVD captures nurses’ self-reported knowledge of recommended EVD care practices through graded endorsement of factually oriented statements; it does not directly verify whether respondents can recall factual answers under objective testing conditions or perform the procedures correctly. Self-reported knowledge therefore represents an important informational component of safe care, but it does not constitute clinical competence. Responses may also be influenced by confidence, familiarity with institutional practices, perceived norms, or response tendencies. Accordingly, the C-SKT-EVD should not be interpreted as a standalone measure of objectively tested factual knowledge or clinical competence. When decisions concerning clinical competence are required, direct observation, simulation-based assessment, structured clinical competency assessment, and evaluation of adherence to local EVD care protocols remain necessary. This distinction is particularly important because psychometric evidence for a self-reported knowledge measure does not, by itself, establish evidence of clinical performance or patient safety (7).

Within its intended scope, the C-SKT-EVD may have practical value for screening self-reported knowledge and assessing educational needs. Its brief format may allow nurse educators to assess baseline knowledge before EVD-related education, identify concepts requiring reinforcement, and reassess knowledge following educational activities. The instrument may also be incorporated as one component of orientation or continuing nursing education for nurses newly assigned to clinical settings involving patients with EVDs. At the nursing-unit level, aggregated item responses may help educators identify commonly misunderstood EVD care concepts and prioritize educational content. Importantly, identified knowledge gaps should not automatically be interpreted as evidence of individual clinical incompetence. Knowledge deficits shared by multiple nurses may instead indicate insufficient emphasis during orientation, inconsistent dissemination of institutional protocols, or limited opportunities for continuing education. Thus, the C-SKT-EVD may serve as one source of information for educational planning and quality-improvement activities rather than as a definitive measure of individual clinical capability.

The person separation findings should be interpreted within the intended purpose of the C-SKT-EVD. The person separation index of 1.86 and Rasch person reliability of 0.78 indicate that the instrument can distinguish approximately two broad levels of the underlying self-reported knowledge trait. These indices provide information about the reproducibility and discriminating capacity of respondent measures within the Rasch framework and should not be interpreted as equivalent to classical internal consistency coefficients. Given the moderate person-level separation and the limited number of items, the instrument provides limited precision for fine-grained individual-level differentiation.

In contrast, the high item separation index (8.48) and item reliability (0.99) indicate that the relative ordering of item difficulties was estimated with high stability in the present sample. This finding supports the stability of the item hierarchy but should not be interpreted as evidence that individual nurses can be classified with the same degree of precision.

Accordingly, the C-SKT-EVD may be more appropriately used for broad assessment of self-reported knowledge of recommended EVD care practices, identification of educational needs, and comparison of knowledge levels at the group level. Its current psychometric evidence does not support the use of the instrument as a standalone tool for high-stakes individual-level pass–fail decisions or clinical competence classification. Importantly, the construct assessed by the C-SKT-EVD should be distinguished from clinical competence. The instrument evaluates nurses’ self-reported knowledge or awareness of recommended EVD care practices rather than directly assessing whether nurses can perform EVD-related procedures correctly in clinical practice. Clinical competence is a broader construct that involves not only factual or declarative knowledge, but also practical skills, clinical judgment, decision-making, and actual performance under clinical conditions. Therefore, C-SKT-EVD scores should be interpreted as one source of information regarding EVD-related knowledge and educational needs and should not be used alone to determine whether a nurse is clinically competent.

Several caveats should be considered when interpreting the present findings. First, the instrument contains only seven items and provides focused rather than comprehensive coverage of EVD care knowledge. The relatively limited targeting at the extreme ends of the latent trait further suggests that additional or complementary items may be required if more precise measurement of very low or very high levels of knowledge is desired. However, expanding the instrument should be guided by theoretical and content considerations rather than by psychometric statistics alone. Second, the present study primarily evaluated the measurement properties of the instrument and did not examine whether C-SKT-EVD scores correspond to actual EVD care performance or patient safety outcomes. Consequently, the current findings should not be interpreted as evidence that higher knowledge scores necessarily indicate better clinical performance or safer patient care. Third, because the five-point agreement format may be influenced by confidence, familiarity with institutional practices, perceived norms, or response tendencies, the present instrument should be considered a measure of self-reported knowledge rather than an objective factual knowledge examination. In addition, the Wright-map results revealed that the item difficulty of the C-SKT-EVD covered a narrow range (−1.0 to +0.8 logits), with insufficient items for the high-ability spectrum. Approximately 20–25% of nurses exhibited ability estimates exceeding the difficulty of the most difficult item, indicating a ceiling effect. This may limit the instrument’s capacity to discriminate nurses with senior professional experience and high-level EVD-care knowledge. Therefore, caution should be exercised when interpreting results if this tool is applied to assess highly experienced specialist nurses or to evaluate knowledge improvement before and after specialized training. Future research could add more difficult items to optimize item-difficulty distribution and further improve item-person targeting. Another limitation concerns the precision and intended interpretation of individual-level scores. The person separation index was 1.86, indicating that the C-SKT-EVD could distinguish approximately two broad levels of the underlying self-reported knowledge trait, but had limited capacity for fine-grained individual differentiation. Therefore, the instrument should not be used to establish high-stakes individual pass–fail decisions or clinical competence classifications. In addition, the C-SKT-EVD assesses self-reported knowledge of recommended EVD care practices rather than direct clinical performance. Clinical competence requires the integration of knowledge with practical skills, clinical judgment, decision-making, and observed performance. Future studies should therefore examine the relationship between C-SKT-EVD scores and objective knowledge tests, structured skills assessments, and observed clinical performance to further establish the instrument’s validity for educational and clinical applications. Finally, although the DIF analyses did not identify substantial DIF, statistically significant differences were observed in selected subgroup comparisons. Complete measurement invariance across all possible nursing subgroups should therefore not be assumed on the basis of the present findings.

An important direction for future research is to establish the relationship between C-SKT-EVD scores and actual clinical performance. Longitudinal studies could examine whether baseline knowledge or changes in C-SKT-EVD scores are associated with objectively observed EVD care quality, including adherence to drainage-system leveling, aseptic practices, monitoring procedures, and recognition and management of abnormal conditions. Such studies could also examine associations with structured clinical competency assessments, adherence to institutional EVD care protocols, and relevant patient safety outcomes. These investigations would help determine whether the self-reported knowledge captured by the C-SKT-EVD is meaningfully associated with subsequent clinical practice and would provide evidence regarding its criterion-related or predictive validity. Importantly, such evidence would complement rather than replace direct assessment of clinical competence. Intervention studies are also needed to determine whether the C-SKT-EVD can contribute to effective continuing nursing education. For example, the instrument could be administered before an EVD-specific educational program to identify individual- or group-level knowledge gaps, followed by targeted teaching and subsequent reassessment. A longitudinal pre–post design or controlled educational intervention could examine whether improvements in C-SKT-EVD scores are accompanied by better knowledge retention, improved adherence to recommended EVD care practices, or improvements in observed clinical performance. If such relationships are demonstrated, the C-SKT-EVD could have greater practical value as part of a structured educational feedback cycle. However, the effectiveness of this approach remains to be established empirically and should not be inferred from the psychometric findings of the present study alone.

## Conclusion

Overall, this study provides preliminary, sample-specific evidence regarding the Rasch measurement properties of the C-SKT-EVD among Chinese nurses. The seven-item instrument demonstrated generally acceptable item functioning, rating-scale performance, measurement targeting, and reliability, supporting its potential use as a brief screening and educational assessment tool for self-reported knowledge of recommended EVD care practices. However, these findings do not establish comprehensive validity across nursing populations or demonstrate objective clinical competence. Further multicenter and longitudinal studies involving diverse nursing populations are warranted to confirm the instrument’s broader applicability and its relationship with objective knowledge and observed clinical performance.

## Limitations

External validity and need for independent validation, Several limitations should be considered regarding the external validity of the findings. First, the participants were recruited from the accessible nursing population within the participating clinical setting(s) using convenience sampling. Therefore, the present sample may not fully represent nurses working in hospitals with different organizational characteristics, geographical regions, levels of experience, or EVD-care practices. Although the C-SKT-EVD demonstrated satisfactory measurement properties in the present sample, the current findings do not establish that the estimated item calibrations, response category functioning, or other psychometric properties will remain stable across different hospitals, regions, or clinical contexts.

Second, the present study did not include an independent external sample for psychometric validation. Accordingly, the reported Rasch measurement properties should be regarded as evidence obtained from the present sample rather than definitive evidence of measurement invariance or generalizability across nursing populations. Independent validation using geographically and institutionally distinct samples is warranted to examine whether the item hierarchy and measurement properties are reproducible across different clinical settings and EVD-care protocols.

Third, the present study used a cross-sectional design and did not evaluate responsiveness to educational interventions or sensitivity to change over time. Therefore, the ability of the C-SKT-EVD to detect changes in nurses’ knowledge following EVD-related education or training remains unknown. Future studies should evaluate responsiveness and sensitivity to change using longitudinal or intervention-based designs. If the instrument is intended for repeated assessment, future research should also examine test–retest reliability and determine whether observed score changes reflect meaningful changes in the underlying construct rather than measurement error.

## Data Availability

The raw data supporting the conclusions of this article will be made available by the authors, without undue reservation.
