# Supplementary material for: Psychometric properties of a short knowledge test to evaluate awareness of external ventricular drain care practices among Chinese nurses: a Rasch model analysis of an efficient 7-item self-reported assessment tool
Source: Front Public Health. 2026 Oct 16;14:1963782. doi: 10.3389/fpubh.2026.1963782 (PMC13719619; doi:10.3389/fpubh.2026.1963782)
Supplement: Supplementary file 1 [file Data_Sheet_1.pdf]

中文版脑室外引流护理实践知晓度简易知识测验量表

A Short Knowledge Test to Evaluate Awareness of External Ventricular Drain Care Practices (Chinese version)

条目<br>**Item**	非常不同意<br>*Strongly disagree*	不同意<br>*Disagree*	中立<br>*Neutral*	同意<br>*Agree*	非常同意<br>*Strongly agree*
脑室外引流装置任何部位断开，都会增加细菌污染风险。<br>*Any disconnection of the EVD system increases the risk of bacterial contamination.*					
脑脊液标本应尽快送检，不得无故拖延；紧急救治患者的情况除外。<br>*Cerebrospinal fluid specimens should be promptly delivered to the laboratory for testing, unless urgent patient care takes priority.*					
应当按照院内规范，通过脑室外引流装置专用采样端口采集脑脊液标本。<br>*Cerebrospinal fluid specimens should be collected from the designated sampling interface of the EVD system in accordance with relevant hospital protocols*					
脑室外引流袋需遵循院内规范进行更换，且不可待引流袋完全充满后再更换<br>*The EVD drainage bag should be replaced in accordance with relevant hospital protocols and before it becomes completely full.*					
需依据院内规范规定的解剖参考标志，对脑室外引流装置进行调平<br>*The EVD system should be leveled according to the anatomical reference point specified in relevant hospital protocols.*					
脑室外引流装置应维持适宜摆放位置，保障引流安全，并最大限度降低污染风险。<br>*The EVD system should be maintained in an appropriate position to ensure safe drainage and reduce the risk of contamination.*					
脑室外引流管路应设置清晰标识，避免与静脉输液管路混淆。<br>*EVD lines should be clearly labeled to prevent confusion with intravenous infusion lines.*					
