# Supplementary material for: Psychometric properties of a short knowledge test to evaluate awareness of external ventricular drain care practices among Chinese nurses: a Rasch model analysis of an efficient 7-item self-reported assessment tool
Source: Front Public Health. 2026 Oct 16;14:1963782. doi: 10.3389/fpubh.2026.1963782 (PMC13719619; doi:10.3389/fpubh.2026.1963782)
Supplement: Supplementary file 2 [file Data_Sheet_2.pdf]

Gender:

PERSON CLASS	DIF MEASURE	DIF S.E.	PERSON CLASS	DIF MEASURE	DIF S.E.	DIF CONTRAST	JOINT S.E.	Welch t	Welch d.f.	Welch Prob.	Mantel-Haenszel Chi-squ	Mantel-Haenszel Prob.	Size CUMLOR	ITEM Number	Name
1	-.97	.08	2	-.97	.13	.00	.16	.00	163	1.000	.0483	.8261	-.07	1	C-item1
1	-.54	.08	2	-.64	.13	.10	.15	.70	162	.4876	1.5797	.2088	.38	2	C-item2
1	-.17	.08	2	-.08	.12	-.09	.14	-.64	164	.5252	.7157	.3976	-.24	3	C-item3
1	.08	.08	2	-.22	.12	.31	.14	2.18	163	.0307	4.0822	.0433	.60	4	C-item4
1	.29	.08	2	.47	.12	-.18	.14	-1.29	164	.1982	1.6183	.2033	-.38	5	C-item5
1	.50	.08	2	.65	.12	-.14	.14	-.99	164	.3247	.8388	.3598	-.26	6	C-item6
1	.79	.08	2	.79	.12	.00	.15	.00	166	1.000	.0417	.8383	-.06	7	C-item7
2	-.97	.13	1	-.97	.08	.00	.16	.00	163	1.000	.0483	.8261	.07	1	C-item1
2	-.64	.13	1	-.54	.08	-.10	.15	-.70	162	.4876	1.5797	.2088	-.38	2	C-item2
2	-.08	.12	1	-.17	.08	.09	.14	.64	164	.5252	.7157	.3976	.24	3	C-item3
2	-.22	.12	1	.08	.08	-.31	.14	-2.18	163	.0307	4.0822	.0433	-.60	4	C-item4
2	.47	.12	1	.29	.08	.18	.14	1.29	164	.1982	1.6183	.2033	.38	5	C-item5
2	.65	.12	1	.50	.08	.14	.14	.99	164	.3247	.8388	.3598	.26	6	C-item6
2	.79	.12	1	.79	.08	.00	.15	.00	166	1.000	.0417	.8383	.06	7	C-item7

Size of Mantel-Haenszel slice: MHSLICE = .010 logits

Professional title:

PERSON CLASS	DIF MEASURE	DIF S.E.	PERSON CLASS	DIF MEASURE	DIF S.E.	DIF CONTRAST	JOINT S.E.	Welch t	Welch d.f.	Welch Prob.	Mantel-Haenszel Chi-squ	Mantel-Haenszel Prob.	Size CUMLOR	ITEM Number	Name
1	-.97	.11	2	-1.04	.13	.07	.17	.44	173	.6636	.2637	.6076	.17	1	C-item1
1	-.97	.11	3	-.97	.16	.00	.19	.00	108	1.000	.2192	.6396	.17	1	C-item1
1	-.97	.11	4	-.45	.32	-.52	.34	-1.55	17	.1404	1.6631	.1972	-1.10	1	C-item1
1	-.60	.10	2	-.60	.12	.00	.15	.03	174	.9777	.0151	.9021	-.04	2	C-item2
1	-.60	.10	3	-.50	.14	-.10	.18	-.56	110	.5777	.2057	.6502	-.16	2	C-item2
1	-.60	.10	4	-.35	.31	-.25	.33	-.75	17	.4606	.1562	.6926	-.30	2	C-item2
1	-.10	.10	2	-.18	.11	.07	.15	.49	175	.6237	.0410	.8396	.06	3	C-item3
1	-.10	.10	3	-.20	.14	.10	.17	.56	110	.5794	.0302	.8621	-.06	3	C-item3
1	-.10	.10	4	-.06	.30	-.04	.32	-.12	17	.9061	.1837	.6682	-.32	3	C-item3
1	-.06	.10	2	.00	.11	-.06	.15	-.39	175	.6952	.7694	.3804	-.28	4	C-item4
1	-.06	.10	3	.09	.14	-.15	.17	-.90	111	.3712	.6177	.4319	-.30	4	C-item4
1	-.06	.10	4	.11	.29	-.17	.31	-.56	17	.5832	.0916	.7621	.24	4	C-item4
1	.35	.10	2	.52	.11	-.17	.15	-1.14	175	.2579	1.3799	.2401	-.38	5	C-item5
1	.35	.10	3	.17	.14	.18	.17	1.02	111	.3108	.7307	.3927	.33	5	C-item5
1	.35	.10	4	-.06	.30	.41	.32	1.29	17	.2131	.0996	.7523	.26	5	C-item5
1	.51	.10	2	.61	.11	-.10	.15	-.69	176	.4918	.0215	.8834	-.04	6	C-item6
1	.51	.10	3	.61	.15	-.11	.18	-.60	110	.5517	.9808	.3220	-.40	6	C-item6
1	.51	.10	4	.11	.29	.40	.31	1.27	18	.2212	.4248	.5146	.42	6	C-item6
1	.90	.11	2	.67	.11	.22	.16	1.40	177	.1639	2.8396	.0920	.57	7	C-item7
1	.90	.11	3	.79	.15	.10	.19	.54	112	.5932	1.6940	.1931	.54	7	C-item7
1	.90	.11	4	.89	.30	.01	.32	.03	18	.9762	.1752	.6755	.31	7	C-item7
2	-1.04	.13	1	-.97	.11	-.07	.17	-.44	173	.6636	.2637	.6076	-.17	1	C-item1
2	-1.04	.13	3	-.97	.16	-.07	.20	-.36	112	.7187	.1004	.7514	-.13	1	C-item1
2	-1.04	.13	4	-.45	.32	-.60	.34	-1.73	19	.1002	.3788	.5382	-.56	1	C-item1
2	-.60	.12	1	-.60	.10	.00	.15	-.03	174	.9777	.0151	.9021	.04	2	C-item2
2	-.60	.12	3	-.50	.14	-.10	.18	-.56	111	.5770	.0371	.8474	-.07	2	C-item2
2	-.60	.12	4	-.35	.31	-.25	.33	-.76	18	.4578	.5691	.4506	-.47	2	C-item2
2	-.18	.11	1	-.10	.10	-.07	.15	-.49	175	.6237	.0410	.8396	-.06	3	C-item3
2	-.18	.11	3	-.20	.14	.02	.18	.13	110	.8983	.0076	.9306	-.03	3	C-item3
2	-.18	.11	4	-.06	.30	-.11	.32	-.35	18	.7327	.4161	.5189	-.39	3	C-item3
2	.00	.11	1	-.06	.10	.06	.15	.39	175	.6952	.7694	.3804	.28	4	C-item4
2	.00	.11	3	.09	.14	-.10	.18	-.55	110	.5863	.1206	.7283	-.13	4	C-item4
2	.00	.11	4	.11	.29	-.12	.31	-.37	18	.7156	.7542	.3852	.62	4	C-item4
2	.52	.11	1	.35	.10	.17	.15	1.14	175	.2579	1.3799	.2401	.38	5	C-item5
2	.52	.11	3	.17	.14	.35	.18	1.95	110	.0533	2.5069	.1133	.62	5	C-item5
2	.52	.11	4	-.06	.30	.58	.32	1.82	18	.0859	4.3100	.0379	1.32	5	C-item5
2	.61	.11	1	.51	.10	.10	.15	.69	176	.4918	.0215	.8834	.04	6	C-item6
2	.61	.11	3	.61	.15	.00	.18	-.02	108	.9879	.0261	.8717	-.06	6	C-item6
2	.61	.11	4	.11	.29	.50	.31	1.59	18	.1286	.1529	.6958	.20	6	C-item6
2	.67	.11	1	.90	.11	-.22	.16	-1.40	177	.1639	2.8396	.0920	-.57	7	C-item7
2	.67	.11	3	.79	.15	-.12	.19	-.64	107	.5251	.2528	.6151	-.20	7	C-item7
2	.67	.11	4	.89	.30	-.21	.32	-.66	18	.5185	2.3920	.1220	-1.06	7	C-item7
3	-.97	.16	1	-.97	.11	.00	.19	.00	108	1.000	.2192	.6396	-.17	1	C-item1
3	-.97	.16	2	-1.04	.13	.07	.20	.36	112	.7187	.1004	.7514	.13	1	C-item1
3	-.97	.16	4	-.45	.32	-.52	.36	-1.47	21	.1576	.5879	.4432	-.63	1	C-item1
3	-.50	.14	1	-.60	.10	.10	.18	.56	110	.5777	.2057	.6502	.16	2	C-item2
3	-.50	.14	2	-.60	.12	.10	.18	.56	111	.5770	.0371	.8474	.07	2	C-item2
3	-.50	.14	4	-.35	.31	-.15	.35	-.44	20	.6670	.0672	.7954	-.23	2	C-item2
3	-.20	.14	1	-.10	.10	-.10	.17	-.56	110	.5794	.0302	.8621	.06	3	C-item3
3	-.20	.14	2	-.18	.11	-.02	.18	-.13	110	.8983	.0076	.9306	.03	3	C-item3
3	-.20	.14	4	-.06	.30	-.13	.33	-.40	20	.6911	.1120	.7379	-.24	3	C-item3
3	.09	.14	1	-.06	.10	.15	.17	.90	111	.3712	.6177	.4319	.30	4	C-item4
3	.09	.14	2	.00	.11	.10	.18	.55	110	.5863	.1206	.7283	.13	4	C-item4
3	.09	.14	4	.11	.29	-.02	.33	-.06	20	.9517	.0044	.9474	-.05	4	C-item4
3	.17	.14	1	.35	.10	-.18	.17	-1.02	111	.3108	.7307	.3927	-.33	5	C-item5
3	.17	.14	2	.52	.11	-.35	.18	-1.95	110	.0533	2.5069	.1133	-.62	5	C-item5
3	.17	.14	4	-.06	.30	.23	.33	.71	20	.4878	.8497	.3566	.92	5	C-item5
3	.61	.15	1	.51	.10	.11	.18	.60	110	.5517	.9808	.3220	.40	6	C-item6
3	.61	.15	2	.61	.11	.00	.18	.02	108	.9879	.0261	.8717	.06	6	C-item6
3	.61	.15	4	.11	.29	.50	.33	1.53	21	.1407	.5570	.4555	.46	6	C-item6
3	.79	.15	1	.90	.11	-.10	.19	-.54	112	.5932	1.6940	.1931	-.54	7	C-item7
3	.79	.15	2	.67	.11	.12	.19	.64	107	.5251	.2528	.6151	.20	7	C-item7
3	.79	.15	4	.89	.30	-.09	.34	-.27	21	.7887	.0013	.9714	-.03	7	C-item7

4	-.45	.32	1	-.97	.11	.52	.34	1.55	17	.1404	1.6631	.1972	1.10	1	C-item1
4	-.45	.32	2	-1.04	.13	.60	.34	1.73	19	.1002	.3788	.5382	.56	1	C-item1
4	-.45	.32	3	-.97	.16	.52	.36	1.47	21	.1576	.5879	.4432	.63	1	C-item1
4	-.35	.31	1	-.60	.10	.25	.33	.75	17	.4606	.1562	.6926	.30	2	C-item2
4	-.35	.31	2	-.60	.12	.25	.33	.76	18	.4578	.5691	.4506	.47	2	C-item2
4	-.35	.31	3	-.50	.14	.15	.35	.44	20	.6670	.0672	.7954	.23	2	C-item2
4	-.06	.30	1	-.10	.10	.04	.32	.12	17	.9061	.1837	.6682	.32	3	C-item3
4	-.06	.30	2	-.18	.11	.11	.32	.35	18	.7327	.4161	.5189	.39	3	C-item3
4	-.06	.30	3	-.20	.14	.13	.33	.40	20	.6911	.1120	.7379	.24	3	C-item3
4	.11	.29	1	-.06	.10	.17	.31	.56	17	.5832	.0916	.7621	-.24	4	C-item4
4	.11	.29	2	.00	.11	.12	.31	.37	18	.7156	.7542	.3852	-.62	4	C-item4
4	.11	.29	3	.09	.14	.02	.33	.06	20	.9517	.0044	.9474	.05	4	C-item4
4	-.06	.30	1	.35	.10	-.41	.32	-1.29	17	.2131	.0996	.7523	-.26	5	C-item5
4	-.06	.30	2	.52	.11	-.58	.32	-1.82	18	.0859	4.3100	.0379	-1.32	5	C-item5
4	-.06	.30	3	.17	.14	-.23	.33	-.71	20	.4878	.8497	.3566	-.92	5	C-item5
4	.11	.29	1	.51	.10	-.40	.31	-1.27	18	.2212	.4248	.5146	-.42	6	C-item6
4	.11	.29	2	.61	.11	-.50	.31	-1.59	18	.1286	.1529	.6958	-.20	6	C-item6
4	.11	.29	3	.61	.15	-.50	.33	-1.53	21	.1407	.5570	.4555	-.46	6	C-item6
4	.89	.30	1	.90	.11	-.01	.32	-.03	18	.9762	.1752	.6755	-.31	7	C-item7
4	.89	.30	2	.67	.11	.21	.32	.66	18	.5185	2.3920	.1220	1.06	7	C-item7
4	.89	.30	3	.79	.15	.09	.34	.27	21	.7887	.0013	.9714	.03	7	C-item7

Size of Mantel-Haenszel slice: MHSLICE = .010 logits

Department:

PERSON CLASS	DIF MEASURE	DIF S.E.	PERSON CLASS	DIF MEASURE	DIF S.E.	DIF CONTRAST	JOINT S.E.	t	Welch d.f.	Prob.	Mantel-Haenszel Chi-squ	Prob.	Size CUMLOR	ITEM Number	Name
1	-1.02	.16	2	-.88	.14	-.14	.21	-.67	101	.5070	.0002	.9898	-.01	1	C-item1
1	-1.02	.16	3	-1.23	.21	.21	.26	.82	74	.4127	.3002	.5837	.31	1	C-item1
1	-1.02	.16	4	-.92	.11	-.10	.19	-.52	108	.6057	.1150	.7346	-.13	1	C-item1
1	-.48	.14	2	-.60	.13	.12	.19	.62	101	.5393	.0072	.9322	-.04	2	C-item2
1	-.48	.14	3	-.45	.18	-.02	.23	-.10	75	.9205	.2786	.5976	-.28	2	C-item2
1	-.48	.14	4	-.63	.11	.16	.18	.89	111	.3747	1.4175	.2338	.44	2	C-item2
1	.12	.14	2	-.27	.13	.38	.19	2.05	101	.0429	3.8028	.0512	.73	3	C-item3
1	.12	.14	3	-.33	.18	.44	.22	1.98	75	.0517	4.9773	.0257	1.13	3	C-item3
1	.12	.14	4	-.15	.10	.26	.17	1.52	110	.1307	1.5460	.2137	.44	3	C-item3
1	.04	.14	2	.07	.12	-.03	.18	-.15	100	.8795	.0204	.8865	.05	4	C-item4
1	.04	.14	3	-.11	.17	.15	.22	.70	76	.4865	.1697	.6804	.19	4	C-item4
1	.04	.14	4	-.04	.10	.08	.17	.47	110	.6406	.0545	.8155	.09	4	C-item4
1	.49	.14	2	.17	.12	.32	.19	1.70	99	.0917	2.0576	.1514	.59	5	C-item5
1	.49	.14	3	.52	.17	-.02	.22	-.11	77	.9126	.7963	.3722	-.46	5	C-item5
1	.49	.14	4	.32	.10	.17	.18	.95	109	.3445	.0631	.8017	.10	5	C-item5
1	.12	.14	2	.69	.13	-.57	.19	-3.06	101	.0028	8.5506	.0035	-1.36	6	C-item6
1	.12	.14	3	.52	.17	-.40	.22	-1.84	76	.0698	.7580	.3839	-.44	6	C-item6
1	.12	.14	4	.70	.11	-.59	.18	-3.33	113	.0012	7.1185	.0076	-1.07	6	C-item6
1	.69	.15	2	.84	.13	-.15	.20	-.76	100	.4508	.5548	.4564	-.35	7	C-item7
1	.69	.15	3	1.03	.18	-.34	.23	-1.46	77	.1490	.8794	.3484	-.44	7	C-item7
1	.69	.15	4	.72	.11	-.03	.19	-.15	110	.8824	.0069	.9337	.03	7	C-item7
2	-.88	.14	1	-1.02	.16	.14	.21	.67	101	.5070	.0002	.9898	.01	1	C-item1
2	-.88	.14	3	-1.23	.21	.35	.25	1.41	77	.1636	.9703	.3246	.52	1	C-item1
2	-.88	.14	4	-.92	.11	.04	.18	.23	134	.8197	.4441	.5052	-.29	1	C-item1
2	-.60	.13	1	-.48	.14	-.12	.19	-.62	101	.5393	.0072	.9322	.04	2	C-item2
2	-.60	.13	3	-.45	.18	-.14	.22	-.64	79	.5270	.0139	.9061	.05	2	C-item2
2	-.60	.13	4	-.63	.11	.04	.17	.23	135	.8221	.7131	.3984	.32	2	C-item2
2	-.27	.13	1	.12	.14	-.38	.19	-2.05	101	.0429	3.8028	.0512	-.73	3	C-item3
2	-.27	.13	3	-.33	.18	.06	.22	.28	78	.7797	.0243	.8762	-.07	3	C-item3
2	-.27	.13	4	-.15	.10	-.12	.16	-.74	136	.4594	1.6686	.1964	-.46	3	C-item3
2	.07	.12	1	.04	.14	.03	.18	.15	100	.8795	.0204	.8865	-.05	4	C-item4
2	.07	.12	3	-.11	.17	.18	.21	.86	78	.3906	.1010	.7507	.16	4	C-item4
2	.07	.12	4	-.04	.10	.11	.16	.68	137	.4976	.0082	.9276	.03	4	C-item4
2	.17	.12	1	.49	.14	-.32	.19	-1.70	99	.0917	2.0576	.1514	-.59	5	C-item5
2	.17	.12	3	.52	.17	-.35	.21	-1.66	78	.1009	1.0621	.3027	-.48	5	C-item5
2	.17	.12	4	.32	.10	-.15	.16	-.95	138	.3450	.3763	.5396	-.22	5	C-item5
2	.69	.13	1	.12	.14	.57	.19	3.06	101	.0028	8.5506	.0035	1.36	6	C-item6
2	.69	.13	3	.52	.17	.17	.21	.81	79	.4183	.4944	.4820	.33	6	C-item6
2	.69	.13	4	.70	.11	-.01	.17	-.08	139	.9347	.6457	.4217	.32	6	C-item6
2	.84	.13	1	.69	.15	.15	.20	.76	100	.4508	.5548	.4564	.35	7	C-item7
2	.84	.13	3	1.03	.18	-.19	.22	-.86	79	.3931	.4800	.4884	-.35	7	C-item7
2	.84	.13	4	.72	.11	.12	.17	.72	138	.4735	.8863	.3465	.35	7	C-item7
3	-1.23	.21	1	-1.02	.16	-.21	.26	-.82	74	.4127	.3002	.5837	-.31	1	C-item1
3	-1.23	.21	2	-.88	.14	-.35	.25	-1.41	77	.1636	.9703	.3246	-.52	1	C-item1
3	-1.23	.21	4	-.92	.11	-.31	.24	-1.33	74	.1887	1.2503	.2635	-.52	1	C-item1
3	-.45	.18	1	-.48	.14	.02	.23	.10	75	.9205	.2786	.5976	.28	2	C-item2
3	-.45	.18	2	-.60	.13	.14	.22	.64	79	.5270	.0139	.9061	-.05	2	C-item2
3	-.45	.18	4	-.63	.11	.18	.21	.86	78	.3913	1.1029	.2936	.44	2	C-item2
3	-.33	.18	1	.12	.14	-.44	.22	-1.98	75	.0517	4.9773	.0257	-1.13	3	C-item3
3	-.33	.18	2	-.27	.13	-.06	.22	-.28	78	.7797	.0243	.8762	.07	3	C-item3
3	-.33	.18	4	-.15	.10	-.18	.20	-.89	78	.3785	1.0921	.2960	-.50	3	C-item3
3	-.11	.17	1	.04	.14	-.15	.22	-.70	76	.4865	.1697	.6804	-.19	4	C-item4
3	-.11	.17	2	.07	.12	-.18	.21	-.86	78	.3906	.1010	.7507	-.16	4	C-item4
3	-.11	.17	4	-.04	.10	-.07	.20	-.37	78	.7140	.1098	.7404	-.15	4	C-item4
3	.52	.17	1	.49	.14	.02	.22	.11	77	.9126	.7962	.3722	.46	5	C-item5
3	.52	.17	2	.17	.12	.35	.21	1.66	78	.1009	1.0621	.3027	.48	5	C-item5
3	.52	.17	4	.32	.10	.19	.20	.97	80	.3331	2.3333	.1266	.76	5	C-item5
3	.52	.17	1	.12	.14	.40	.22	1.84	76	.0698	.7580	.3839	.44	6	C-item6
3	.52	.17	2	.69	.13	-.17	.21	-.81	79	.4183	.4944	.4820	-.33	6	C-item6
3	.52	.17	4	.70	.11	-.19	.20	-.92	82	.3602	.5693	.4505	-.33	6	C-item6
3	1.03	.18	1	.69	.15	.34	.23	1.46	77	.1490	.8794	.3484	.44	7	C-item7
3	1.03	.18	2	.84	.13	.19	.22	.86	79	.3931	.4800	.4884	.35	7	C-item7
3	1.03	.18	4	.72	.11	.31	.21	1.49	80	.1410	1.0887	.2968	.40	7	C-item7

4	-.92	.11	1	-1.02	.16	.10	.19	.52	108	.6057	.1150	.7346	.13	1	C-item1
4	-.92	.11	2	-.88	.14	-.04	.18	-.23	134	.8197	.4441	.5052	.29	1	C-item1
4	-.92	.11	3	-1.23	.21	.31	.24	1.33	74	.1887	1.2503	.2635	.52	1	C-item1
4	-.63	.11	1	-.48	.14	-.16	.18	-.89	111	.3747	1.4175	.2338	-.44	2	C-item2
4	-.63	.11	2	-.60	.13	-.04	.17	-.23	135	.8221	.7131	.3984	-.32	2	C-item2
4	-.63	.11	3	-.45	.18	-.18	.21	-.86	78	.3913	1.1029	.2936	-.44	2	C-item2
4	-.15	.10	1	.12	.14	-.26	.17	-1.52	110	.1307	1.5460	.2137	-.44	3	C-item3
4	-.15	.10	2	-.27	.13	.12	.16	.74	136	.4594	1.6686	.1964	.46	3	C-item3
4	-.15	.10	3	-.33	.18	.18	.20	.89	78	.3785	1.0921	.2960	.50	3	C-item3
4	-.04	.10	1	.04	.14	-.08	.17	-.47	110	.6406	.0545	.8155	-.09	4	C-item4
4	-.04	.10	2	.07	.12	-.11	.16	-.68	137	.4976	.0082	.9276	-.03	4	C-item4
4	-.04	.10	3	-.11	.17	.07	.20	.37	78	.7140	.1098	.7404	.15	4	C-item4
4	.32	.10	1	.49	.14	-.17	.18	-.95	109	.3445	.0631	.8017	-.10	5	C-item5
4	.32	.10	2	.17	.12	.15	.16	.95	138	.3450	.3763	.5396	.22	5	C-item5
4	.32	.10	3	.52	.17	-.19	.20	-.97	80	.3331	2.3333	.1266	-.76	5	C-item5
4	.70	.11	1	.12	.14	.59	.18	3.33	113	.0012	7.1185	.0076	1.07	6	C-item6
4	.70	.11	2	.69	.13	.01	.17	.08	139	.9347	.6457	.4217	-.32	6	C-item6
4	.70	.11	3	.52	.17	.19	.20	.92	82	.3602	.5693	.4505	.33	6	C-item6
4	.72	.11	1	.69	.15	.03	.19	.15	110	.8824	.0069	.9337	-.03	7	C-item7
4	.72	.11	2	.84	.13	-.12	.17	-.72	138	.4735	.8863	.3465	-.35	7	C-item7
4	.72	.11	3	1.03	.18	-.31	.21	-1.49	80	.1410	1.0887	.2968	-.40	7	C-item7

Size of Mantel-Haenszel slice: MHSLICE = .010 logits

Working years:

PERSON CLASS	DIF MEASURE	DIF S.E.	PERSON CLASS	DIF MEASURE	DIF S.E.	DIF CONTRAST	JOINT S.E.	Welch t	Welch d.f.	Welch Prob.	Mantel-Haenszel Chi-squ	Mantel-Haenszel Prob.	Size CUMLOR	ITEM Number	Name
1	-1.02	.10	2	-.97	.13	-.05	.16	-.31	179	.7567	.0415	.8386	-.07	1	C-item1
1	-1.02	.10	3	-.83	.16	-.19	.19	-1.02	106	.3109	.1651	.6845	-.16	1	C-item1
1	-.57	.10	2	-.54	.11	-.02	.15	-.16	181	.8722	.0596	.8071	-.08	2	C-item2
1	-.57	.10	3	-.59	.15	.02	.18	.12	105	.9032	.0022	.9629	.02	2	C-item2
1	-.11	.09	2	-.21	.11	.10	.14	.72	182	.4708	.6669	.4141	.25	3	C-item3
1	-.11	.09	3	-.12	.14	.01	.17	.05	106	.9592	.1254	.7233	-.13	3	C-item3
1	.00	.09	2	-.08	.11	.08	.14	.56	182	.5789	.0508	.8218	.07	4	C-item4
1	.00	.09	3	.15	.14	-.15	.17	-.89	107	.3765	.1187	.7305	-.13	4	C-item4
1	.31	.10	2	.50	.11	-.19	.15	-1.30	183	.1936	2.0021	.1571	-.46	5	C-item5
1	.31	.10	3	.15	.14	.16	.17	.95	108	.3448	.3193	.5720	.21	5	C-item5
1	.48	.10	2	.70	.11	-.22	.15	-1.49	182	.1376	2.7790	.0955	-.48	6	C-item6
1	.48	.10	3	.41	.14	.07	.17	.41	109	.6825	.1759	.6749	-.17	6	C-item6
1	.93	.11	2	.61	.11	.32	.15	2.08	186	.0386	6.1579	.0131	.79	7	C-item7
1	.93	.11	3	.86	.15	.07	.19	.35	110	.7274	1.1293	.2879	.41	7	C-item7
2	-.97	.13	1	-1.02	.10	.05	.16	.31	179	.7567	.0415	.8386	.07	1	C-item1
2	-.97	.13	3	-.83	.16	-.14	.20	-.70	109	.4864	.0488	.8252	-.10	1	C-item1
2	-.54	.11	1	-.57	.10	.02	.15	.16	181	.8722	.0596	.8071	.08	2	C-item2
2	-.54	.11	3	-.59	.15	.05	.19	.24	107	.8085	.0289	.8650	.07	2	C-item2
2	-.21	.11	1	-.11	.09	-.10	.14	-.72	182	.4708	.6669	.4141	-.25	3	C-item3
2	-.21	.11	3	-.12	.14	-.10	.18	-.53	107	.5973	.0581	.8095	-.09	3	C-item3
2	-.08	.11	1	.00	.09	-.08	.14	-.56	182	.5789	.0508	.8218	-.07	4	C-item4
2	-.08	.11	3	.15	.14	-.23	.18	-1.30	107	.1967	1.7818	.1819	-.54	4	C-item4
2	.50	.11	1	.31	.10	.19	.15	1.30	183	.1936	2.0021	.1571	.46	5	C-item5
2	.50	.11	3	.15	.14	.35	.18	1.97	107	.0510	2.5548	.1100	.65	5	C-item5
2	.70	.11	1	.48	.10	.22	.15	1.49	182	.1376	2.7790	.0955	.48	6	C-item6
2	.70	.11	3	.41	.14	.29	.18	1.62	108	.1087	.8133	.3671	.35	6	C-item6
2	.61	.11	1	.93	.11	-.32	.15	-2.08	186	.0386	6.1579	.0131	-.79	7	C-item7
2	.61	.11	3	.86	.15	-.26	.19	-1.37	105	.1748	1.1649	.2805	-.42	7	C-item7
3	-.83	.16	1	-1.02	.10	.19	.19	1.02	106	.3109	.1651	.6845	.16	1	C-item1
3	-.83	.16	2	-.97	.13	.14	.20	.70	109	.4864	.0488	.8252	.10	1	C-item1
3	-.59	.15	1	-.57	.10	-.02	.18	-.12	105	.9032	.0022	.9629	-.02	2	C-item2
3	-.59	.15	2	-.54	.11	-.05	.19	-.24	107	.8085	.0289	.8650	-.07	2	C-item2
3	-.12	.14	1	-.11	.09	-.01	.17	-.05	106	.9592	.1254	.7233	.13	3	C-item3
3	-.12	.14	2	-.21	.11	.10	.18	.53	107	.5973	.0581	.8095	.09	3	C-item3
3	.15	.14	1	.00	.09	.15	.17	.89	107	.3765	.1187	.7305	.13	4	C-item4
3	.15	.14	2	-.08	.11	.23	.18	1.30	107	.1967	1.7818	.1819	.54	4	C-item4
3	.15	.14	1	.31	.10	-.16	.17	-.95	108	.3448	.3193	.5720	-.21	5	C-item5
3	.15	.14	2	.50	.11	-.35	.18	-1.97	107	.0510	2.5548	.1100	-.65	5	C-item5
3	.41	.14	1	.48	.10	-.07	.17	-.41	109	.6825	.1759	.6749	.17	6	C-item6
3	.41	.14	2	.70	.11	-.29	.18	-1.62	108	.1087	.8133	.3671	-.35	6	C-item6
3	.86	.15	1	.93	.11	-.07	.19	-.35	110	.7274	1.1293	.2879	-.41	7	C-item7
3	.86	.15	2	.61	.11	.26	.19	1.37	105	.1748	1.1649	.2805	.42	7	C-item7

Size of Mantel-Haenszel slice: MHSLICE = .010 logits
